# APRI and FIB-4 in the evaluation of liver fibrosis in chronic hepatitis C patients stratified by AST level

**DOI:** 10.1371/journal.pone.0199760

**Published:** 2018-06-28

**Authors:** Yi-Hao Yen, Fang-Ying Kuo, Kwong-Ming Kee, Kuo-Chin Chang, Ming-Chao Tsai, Tsung-Hui Hu, Sheng-Nan Lu, Jing-Houng Wang, Chao-Hung Hung, Chien-Hung Chen

**Affiliations:** 1 Division of Hepatogastroenterology, Department of Internal Medicine, Kaohsiung Chang Gung Memorial Hospital and Chang Gung University College of Medicine, Kaohsiung, Taiwan; 2 Department of Pathology, Kaohsiung Chang Gung Memorial Hospital and Chang Gung University College of Medicine, Kaohsiung, Taiwan; Medizinische Fakultat der RWTH Aachen, GERMANY

## Abstract

**Background and aim:**

The aspartate aminotransferase (AST)-to-platelet ratio index (APRI) and fibrosis-4 index (FIB-4) are commonly used compound surrogates for advanced fibrosis in chronic hepatitis C (CHC) patients. However, the use of APRI and FIB-4 entails a risk of overestimating the fibrosis stage due to the impact of necroinflammatory activity on transaminases. We sought to investigate the optimal cutoff values of the two compound surrogates for predicting cirrhosis stratified by AST level.

**Methods:**

This retrospective study enrolled 1716 treatment-naive CHC patients who underwent liver biopsy prior to interferon therapy from 1997–2010. Fibrosis was scored according to the modified Knodell classification. The upper limit for normal AST in our hospital is 37 IU/L. We stratified the enrolled patients into the categories of AST≤37 IU/L (N = 132), 37<AST≤74, (N = 501), 74<AST≤148 IU/L (N = 737), and AST>148 IU/L (N = 346).

**Results:**

436 patients had cirrhosis (F4). The area under receiver operating characteristic (AUROC) analysis results distinguishing cirrhosis (F4) from non-cirrhosis (F0–F3) were 0.81 for APRI and 0.85 for FIB-4 in patients with AST≤37 IU/L; 0.71 for APRI and 0.72 for FIB-4 in patients with 37<AST≤74IU/L; 0.72 for APRI and 0.73 for FIB-4 in patients with 74<AST≤148 IU/L; and 0.68 for APRI and 0.70 for FIB-4 in patients with AST>148 IU/L. The optimal cutoff values of APRI and FIB-4 for the diagnosis of cirrhosis were 0.6 and 1.4, respectively, in patients with AST≤37 IU/L; 1.1 and 2.2, respectively, in patients with 37<AST≤74 IU/L; 2.2 and 3.4, respectively, in patients with 74<AST≤148 IU/L; and 3.4 and 5.5, respectively, in patients with AST>148 IU/L.

**Conclusions:**

We provide optimal cutoff values of both APRI and FIB-4 to predict cirrhosis stratified by AST levels, which should be more feasible compared with the single cutoff values proposed in previous studies.

## Introduction

Hepatitis C virus (HCV) infection is one of the common causes of chronic liver disease worldwide [1]. The long-term impacts of HCV infection are highly variable, ranging from minimal histological changes to cirrhosis and hepatocellular carcinoma (HCC). The number of chronically infected patients worldwide is estimated to be about 180 million [2].

The advent of the new direct-acting antivirals (DAAs) for the treatment of HCV has substantially increased sustained virological response (SVR) rates. However, the main goal of their use is to increase survival and quality of life by modifying the natural history of the infection (that is, by interrupting the sequence of fibrosis to cirrhosis and HCC) beyond eradication of the virus [3]. Nevertheless, universal access to these treatments has not been possible due to their higher costs compared with older therapies. Liver fibrosis is the essential concern in the treatment of HCV infection.

Data indicating that even moderate fibrosis may be associated with worse outcomes in the context of HCV infection have come from the HALT-C (Hepatitis C Antiviral Long-Term Treatment Against Cirrhosis) trial, which examined the effects of maintenance therapy with pegylated interferon among patients with chronic HCV infection and fibrosis. The 6-year cumulative incidence of a first clinical event was 5.6% for Ishak F2, 16.1% for Ishak F3, 19.3% for Ishak F4, 37.8% for Ishak F5, and 49.3% for Ishak F6 [4, 5]. Furthermore, the EASL guidelines recommend that treatment should be considered without delay in patients with significant fibrosis or cirrhosis (i.e., a METAVIR score of F2, F3, or F4) [6].

Numerous serologic tests have been developed to detect liver fibrosis. The aspartate aminotransferase (AST)-to-platelet ratio index (APRI) and fibrosis-4 index (FIB-4) scoring are the most widely used compound surrogates for advanced fibrosis. However, the use of the APRI and FIB-4 entails a risk of overestimating the fibrosis stage due to the impact of necroinflammatory activity on transaminases [7]. The aim of this study, then, was to investigate the optimal cutoff values of APRI and FIB-4 in the evaluation of liver fibrosis in treatment-naive chronic hepatitis C (CHC) patients stratified by AST level.

## Patients and methods

### Patients

This retrospective cohort study included consecutive adult patients with CHC who had undergone percutaneous liver biopsy prior to interferon therapy at Kaoshiung Chang Gung Memorial Hospital from December 1997 to October 2010. The diagnosis of CHC was established by the presence of HCV RNA using polymerase chain reaction assays. Patients with the following conditions were excluded from the study: the presence of other causes of liver disease, HCC, prior interferon therapy, human immunodeficiency virus (HIV) co-infection, and liver transplantation prior to liver biopsy. The diagnosis of HCC was based on recommended criteria [8, 9].

### Demographic and laboratory data

Clinical information about these patients obtained from electronic medical records and hard-copy clinical charts was reviewed by one investigator (Y.H.Y.) to assess each patient’s eligibility for the study. The demographics and laboratory variables of each patient were recorded. Except for HCV genotype, only laboratory results obtained within 3 months from the date of the liver biopsy were used for this study. If more than one set of laboratory test results was available, the results obtained closest to the time of the biopsy were used.

### Liver histology evaluation

Each patient received an echo-guided percutaneous liver biopsy from the right hepatic lobe conducted using a 16-gauge Bard^®^ Max-core^®^ biopsy instrument. The sampling tissues were fixed with formalin, embedded with paraffin, and stained with H&E and reticulin silver “i.e., the Masson trichrome method was used”. Histology was reported by three pathologists, all of whom had no knowledge of the clinical characteristics of the study subjects. The degree of liver necroinflammation in each patient was calculated by Histology Activity Index scores [10]. Meanwhile, the degree of liver fibrosis in each patient was graded and staged according to the modified Knodell histology index [11].

### APRI and FIB-4 formula

The FIB-4 values were calculated automatically using the formula age (years) × AST [U/l]/(platelets [10^9^/l] × (ALT [U/l])^1/2^), in which the age of the patient was the age at the time of the liver biopsy [12]. The APRI values were calculated using the formula “AST/upper limit of normal]/platelet count [10^9^/L]” × 100 [13]. The upper limit of normal of AST in our hospital is 37 IU/L.

All the procedures used in the study were in accordance with the ethical standards of the responsible committees on human experimentation (institutional and national) and with the Helsinki Declaration of 1975, as revised in 2008. This study was approved by the Institutional Review Board of Kaohsiung Chang Gung Memorial Hospital (IRB number: 201601607B0). The requirement for informed consent was waived by the IRB. The data were analyzed anonymously.

### Statistical analysis

The comparisons of the patient characteristics at different AST levels were estimated using the one-way ANOVA or chi-square test as appropriate. The diagnostic accuracies of APRI and FIB-4 for predicting different stages of liver fibrosis were expressed as area under receiver operating characteristic (AUROC) curves. An AUROC of 1.0 indicates perfect discrimination, while an AUROC of 0.5 indicates a lack of discrimination. A value of 0.90–1.0 has been classified as excellent, a value of 0.80–0.90 as good, a value of 0.70–0.80 as fair, and a value < 0.70 as poor [14, 15]. The AUROC curves of the APRI and FIB-4 for predicting fibrosis stage (≥2, ≥3, and = 4) were also calculated, and the significance of the statistical difference between the curves was examined [16]. The respective APRI and FIB-4 values with the highest Youden’s index (sensitivity + specificity—1) yielded by the ROC analysis in terms of diagnostic accuracies for fibrosis stage were selected as the best cutoff values. The diagnostic accuracies of APRI and FIB-4 for predicting different stages of liver fibrosis using the best estimated APRI and FIB-4 cutoff values were defined as AUROC_*cutoff*_ values. Moreover, a prediction model validation was performed using a bootstrap resampling method in order to test the robustness of the AUROC_*cutoff*_ values. The bootstrap resampling process generates 1000 replications for each validation, and the resulting bootstrap AUROC_*cutoff*_ values were reported. The correlations of APRI and FIB-4 with clinical variables were calculated with Pearson correlation coefficients. All of the statistical analyses were performed using the Stata version 11.0 statistical software (Stata Corp., College Station, TX, 2009). All P values less than .05 were considered statistically significant.

## Results

### Characteristics of the patients

From December 1997 to October 2010, 2055 percutaneous liver biopsies were performed on patients with CHC at our institution. A total of 339 of those patients were excluded from this study: 128 had a hepatitis B virus (HBV) co-infection, 40 had a history of alcoholism, 6 had an HIV co-infection, 102 had received prior interferon therapy, 32 had a history of HCC, and 31 had missing AST, alanine-aminotransferase (ALT), or platelet count data within the 3 months prior to liver biopsy. Thus, a cohort comprising 1716 patients was analyzed in the present study. The characteristics of these patients are shown in Table 1. The mean age was 52.5 years; 53.2% of the patients were male; 46.7% of the patients were genotype 1; 48.5% of the patients were genotype 2; the mean necroinflammation score was 7.3; the median AST level was 92 IU/L (range: 11–640 IU/L); the median ALT level was 134 IU/L (range: 9–1253 IU/L); 680 (39.63%) of the patients had thrombocytopenia (<150 10^9^/L); and the platelet count range was 19–453 (10^9^/L). Furthermore, 163 (9.5%) of the patients had a fibrosis score of 2; 309 (18.01%) of the patients had a fibrosis score of 3; and 436 (25.41%) of the patients had cirrhosis.

**Table 1:** Comparsion of patients according to AST level.

Variable	All, N = 1716	AST<37, N = 132	37<AST≤74, N = 501	74<AST≤148 IU/L, N = 737	AST>148 IU/L, N = 346	*P*
Age (years)	52.5±11.6	46.7±12	50.1±12.2	53.9±10.7	55.3±10.7	<0.001
Male gender, N (*%*)	913 (53.2%)	65 (49.2%)	294 (58.7%)	383 (52.0%)	171 (49.4%)	0.025
Genotype, N (*%*)						0.099
1	728 (46.7%)	47 (40.2%)	230 (49.8%)	322 (48.6%)	129 (40.6%)	
2	756 (48.5%)	59 (50.4%)	211 (45.7%)	312 (47.1%)	174 (54.7%)	
Mixed	50 (3.2%)	8 (6.8%)	13 (2.8%)	19 (2.9%)	10 (3.1%)	
Others	26 (1.6%)	3 (2.6%)	8 (1.7%)	10 (1.5%)	5 (1.6%)	
Missing data	156 (9.1%)	15 (11.4%)	39 (7.8%)	74 (10.0%)	28 (8.1%)	
BMI (kg/m^2^)	24.6±3.5	23.8±3.4	24.7±3.4	24.7±3.6	24.7±3.5	0.09
Missing data	181 (10.6%)	22 (16.7%)	47 (9.4%)	70 (9.5%)	42 (12.1%)	
Necroinflammation score	7.3±2.3	5.1±2.0	6.6±2.2	7.7±2.1	8.2±2.3	<0.001
Fibrosis						<0.001
0	325 (18.9%)	77 (58.3%)	128 (25.5%)	73 (9.9%)	47 (13.6%)	
1	483 (28.2%)	23 (17.4%)	178 (35.5%)	209 (28.4%)	73 (21.1%)	
2	163 (9.5%)	9 (6.8%)	48 (9.6%)	78 (10.6%)	28 (8.1%)	
3	309 (18.0%)	17 (12.9%)	65 (13%)	150 (20.4%)	77 (22.3%)	
4	436 (25.4%)	6 (4.5%)	82 (16.4%)	227 (30.8%)	121 (35.0%)	
HCV RNA >600,000 IU/ml, N (*%*)	262 (27.3%)	37 (38.9%)	92 (31.2%)	81 (21.2%)	52 (27.8%)	0.001
Only qualitative HCV RNA was available, N (*%*)	756 (44.1%)	37 (28%)	206 (41.1%)	354 (48.0%)	159 (46.0%)	
ALT (IU/L)	134 (93–205.5)	44 (32–57)	92 (75–111)	152 (120–195)	273.5 (209–366)	<0.001
Platelet (10^9^/L) <150	680 (39.6%)	24 (18.2%)	152 (30.3%)	341 (46.3%)	163 (47.1%)	<0.001
Bilirubin (mg/dL)	0.9±0.6	0.9±0.5	0.9±0.5	0.9±0.6	1.0±0.8	0.75
Albumin (mg/dL)	3.9±0.6	4.0±0.4	4.2±0.4	3.9±0.6	3.7±0.6	<0.001
INR	1.0±0.1	1.1±0.1	1.0±0.1	1.0±0.1	1.1±0.1	0.04

Data were expressed as mean ± SD or median (interquantile). BMI, body mass index; AST, Aspartate Aminotransferase; ALT, Alanine Aminotransferase; INR, international normalized ratio

### Comparison of patients according to AST levels

A comparison of the patients according to AST levels is shown in Table 1. In comparison to those with AST ≤148 IU/L, those with AST> 148 IU/L were older, had higher necroinflammation scores, had higher ALT levels, had lower albumin levels, and included higher proportions of patients who had thrombocytopenia and cirrhosis. In comparison to those with AST ≥37 IU/L, those with AST <37 IU/L were younger, had lower necroinflammation scores, had lower ALT levels, and included lower proportions of patients who had thrombocytopenia and cirrhosis.

### Diagnostic accuracies of APRI and FIB-4 for predicting liver fibrosis in all patients

The AUROCs of APRI and FIB-4 for the diagnosis of ≥F2, ≥ F3, and F4 were 0.68 and 0.70, 0.68 and 0.73, and 0.70 and 0.73, respectively. The optimal cutoff values of APRI and FIB-4 for the diagnosis of ≥F2, ≥ F3, and F4 were 1.4 and 2.9, 1.6 and 2.9, and 2.2 and 3.1, respectively (Table 2).

**Table 2:** Diagnostic accuracies of APRI and FIB-4 for predicting liver fibrosis (all patients, N = 1716).

Index	AUROC_*cutoff*_	cutoff	sensitivity_*cutoff*_	specificity_*cutoff*_	PPV_*cutoff*_	NPV_*cutoff*_	Sensitivity + Specificity-1
To predict fibrosis ≥2							
APRI	0.68 (0.66–0.70)	1.4	72.4%	63.2%	68.9%	67.1%	35.6%
FIB-4	0.70 (0.68–0.72)	2.9	62.7%	78.0%	76.2%	65.0%	40.6%
To predict fibrosis ≥3							
APRI	0.68 (0.66–0.70)	1.6	67.1%	68.8%	62.3%	62.3%	36.6%
FIB-4	0.73 (0.71–0.75)	2.9	69.1%	76.1%	68.9%	76.3%	45.2%
To predict fibrosis = 4							
APRI	0.70 (0.68–0.73)	2.2	64.9%	75.4%	47.3%	86.3%	40.3%
FIB-4	0.73 (0.70–0.75)	3.1	72.0%	73.4%	47.9%	88.5%	46.6%

APRI, aspartate aminotransferase (AST)- to-platelet ratio index; FIB-4, fibrosis index based on the four factors; AUROC, area under receiver operating characteristic

### Diagnostic accuracies of APRI and FIB-4 for predicting fibrosis in patients with AST≤ 37 IU/L

The bootstrap AUROCs of APRI and FIB-4 for the diagnosis of ≥F2, ≥ F3, and F4 were 0.65 and 0.67, 0.73 and 0.77, and 0.81 and 0.85, respectively, in patients with AST≤37 IU/L. The optimal cutoff values of APRI and FIB-4 for the diagnosis of ≥F2, ≥ F3, and F4 were 0.5 and 1.6, 0.6 and 1.4, and 0.6 and 1.4, respectively, in patients with AST≤37 IU/L (Table 3).

**Table 3:** Diagnostic accuracies of APRI and FIB-4 for predicting liver fibrosis (AST≤37 IU/L, N = 132).

Index	AUROC_*cutoff*_	Bootstrap AUROC_*cutoff*_	cutoff	sensitivity_*cutoff*_	specificity_*cutoff*_	PPV_*cutoff*_	NPV_*cutoff*_	Sensitivity + Specificity-1
To predict fibrosis ≥2								
APRI	0.65 (0.56–0.75)	0.65 (0.56–0.75)	0.5	46.9%	84.0%	48.4%	83.2%	33.9%
FIB-4	0.67 (0.58–0.76)	0.67(0.57–0.77)	1.6	46.9%	87.0%	53.6%	83.7%	43.4%
To predict fibrosis ≥3								
APRI	0.73 (0.62–0.83)	0.73 (0.62–0.84)	0.6	60.9%	84.4%	45.2%	91.1%	45.3%
FIB-4	0.77 (0.67–0.86)	0.767 (0.67–0.86)	1.4	78.3%	75.2%	40.0%	94.3%	53.5%
To predict fibrosis = 4								
APRI	0.81 (0.65–0.98)	0.81 (0.64–1.0)	.6	83.3%	79.4%	16.1%	99.0%	62.7%
FIB-4	0.85 (0.81–0.89)	0.85 (0.81–0.89)	1.4	100.0%	69.1%	13.3%	100.0%	69.1%

APRI, aspartate aminotransferase (AST)- to-platelet ratio index; FIB-4, fibrosis index based on the four factors; AUROC, area under receiver operating characteristic

### Diagnostic accuracies of APRI and FIB-4 for predicting liver fibrosis in patients with 37<AST≤74 IU/L

The bootstrap AUROCs of APRI and FIB-4 for the diagnosis of ≥F2, ≥ F3, and F4 were 0.65 and 0.65, 0.69 and 0.70, and 0.71 and 0.72, respectively, in patients with 37<AST≤ 74 IU/L. The optimal cutoff values of APRI and FIB-4 for the diagnosis of ≥F2, ≥ F3, and F4 were 1.1 and 2.2, 2.2 and 1.0, and 1.1 and 2.2, respectively, in patients with 37<AST≤74 IU/L (Table 4).

**Table 4:** Diagnostic accuracies of APRI and FIB-4 for predicting liver fibrosis (37<AST≤74, N = 501).

Index	AUROC_*cutoff*_	Bootstrap AUROC_*cutoff*_	cutoff	sensitivity_*cutoff*_	specificity_*cutoff*_	PPV_*cutoff*_	NPV_*cutoff*_	Sensitivity + Specificity-1
To predict fibrosis ≥2								
APRI	0.65 (0.60–0.69)	0.65 (0.60–0.69)	1.1	50.8%	78.4%	60.0%	71.4%	29.2%
FIB-4	0.65 (0.61–0.69)	0.65 (0.60–0.69)	2.2	53.3%	76.1%	58.8%	71.9%	29.5%
To predict fibrosis ≥3								
APRI	0.69 (0.64–0.73)	0.69 (0.64–0.73)	1.0	59.2%	78.0%	52.7%	82.1%	37.3%
FIB-4	0.70(0.65–0.74)	0.70(0.65–0.74)	2.2	63.3%	76.3%	52.5%	83.3%	39.5%
To predict fibrosis = 4								
APRI	0.71 (0.66–0.77)	0.71 (0.66–0.77)	1.1	68.3%	74.0%	33.9%	92.3%	42.3%
FIB-4	0.72 (0.67–0.77)	0.719 (0.67–0.77)	2.2	72.0%	71.8%	33.3%	92.9%	43.8%

APRI, aspartate aminotransferase (AST)- to-platelet ratio index; FIB-4, fibrosis index based on the four factors; AUROC, area under receiver operating characteristic

### Diagnostic accuracies of APRI and FIB-4 for predicting liver fibrosis in patients with 74<AST≤148 IU/L

The bootstrap AUROCs of APRI and FIB-4 for the diagnosis of ≥ F2, ≥ F3, and F4 were 0.66 and 0.69, 0.69 and 0.71, and 0.72 and 0.73, respectively, in patients with 74<AST≤148 IU/L. The optimal cutoff values of APRI and FIB-4 for the diagnosis of ≥F2, ≥ F3, and F4 were 1.9 and 3.0, 2.2 and 3.2, and 2.2 and 3.4, respectively, in patients with 74<AST≤148 IU/L (Table 5).

**Table 5:** Diagnostic accuracies of APRI and FIB-4 for predicting liver fibrosis (74<AST≤148 IU/L, N = 737).

Index	AUROC_*cutoff*_	Bootstrap AUROC_*cutoff*_	cutoff	sensitivity_*cutoff*_	specificity_*cutoff*_	PPV_*cutoff*_	NPV_*cutoff*_	Sensitivity + Specificity-1
To predict fibrosis ≥2								
APRI	0.66 (0.63–0.69)	0.66 (0.63–0.69)	1.9	51.9%	79.8%	80.6%	50.7%	33.3%
FIB-4	0.69 (0.65–0.72)	0.69 (0.65–0.72)	3.0	62.2%	74.8%	79.9%	55.1%	37.0%
To predict fibrosis ≥3								
APRI	0.69 (0.66–0.72)	0.69 (0.66–0.72)	2.2	55.4%	82.5%	76.8%	63.9%	37.9%
FIB-4	0.71 (0.68–0.74)	0.71 (0.69–0.76)	3.2	64.7%	76.9%	74.6%	67.6%	41.7%
To predict fibrosis = 4								
APRI	0.72 (0.68–0.76)	0.72 (0.69–0.76)	2.2	67.4%	76.7%	56.3%	84.1%	44.1%
FIB-4	0.73 (0.69–0.76)	0.73 (0.69–0.76)	3.4	70.0%	74.9%	55.4%	84.9%	45.7%

APRI, aspartate aminotransferase (AST)- to-platelet ratio index; FIB-4, fibrosis index based on the four factors; AUROC, area under receiver operating characteristic

### Diagnostic accuracies of APRI and FIB-4 for predicting liver fibrosis in patients with AST>148 IU/L

The bootstrap AUROCs of APRI and FIB-4 for the diagnosis of ≥F2, ≥ F3, and F4 were 0.68 and 0.68, 0.68 and 0.70, and 0.68 and 0.70, respectively, in patients with AST>148 IU/L. The optimal cutoff values of APRI and FIB-4 for the diagnosis of ≥F2, ≥ F3, and F4 were 3.4 and 3.4, 3.4 and 5.2, and 3.4 and 5.5, respectively, in patients with AST>148 IU/L (Table 6).

**Table 6:** Diagnostic accuracies of APRI and FIB-4 for predicting liver fibrosis (AST>148 IU/L, N = 346).

Index	AUROC_*cutoff*_	Bootstrap AUROC_*cutoff*_	cutoff	sensitivity_*cutoff*_	specificity_*cutoff*_	PPV_*cutoff*_	NPV_*cutoff*_	Sensitivity + Specificity-1
To predict fibrosis ≥2								
APRI	0.68 (0.62–0.73)	0.676 (0.62–0.73)	3.4	65.0%	70.0%	80.3%	51.5%	36.5%
FIB-4	0.68 (0.63–0.73)	0.68 (0.63–0.73)	3.4	79.7%	56.7%	77.6%	59.7%	38.6%
To predict fibrosis ≥3								
APRI	0.68 (0.63–0.73)	0.68 (0.63–0.73)	3.4	68.2%	67.6%	73.8%	61.4%	38.3%
FIB-4	0.70 (0.65–0.74)	0.70 (0.65–0.74)	5.2	58.6%	80.4%	80.0%	59.2%	39.2%
To predict fibrosis = 4								
APRI	0.68 (0.64–0.73)	0.68 (0.64–0.73)	3.4	76.9%	60.0%	50.8%	82.8%	40.4%
FIB-4	0.70 (0.65–0.75)	0.70 (0.65–0.75)	5.5	61.2%	79.1%	61.2%	79.1%	43.5%

APRI, aspartate aminotransferase (AST)- to-platelet ratio index; FIB-4, fibrosis index based on the four factors; AUROC, area under receiver operating characteristic

### Comparison of diagnostic accuracy between APRI and FIB-4

A comparison of the ROC of APRI vs. that of FIB-4 to predict fibrosis ≥2 in patients with AST≤37 IU/L is shown in S1 Fig, P = 0.11. A comparison of the ROC of APRI vs. that of FIB-4 to predict fibrosis ≥2 in patients with 37<AST≤74 IU/L is shown in S2 Fig, P = 0.86. A comparison of the ROC of APRI vs. that of FIB-4 to predict fibrosis ≥2 in patients with 74<AST≤148 IU/L is shown in S3 Fig, P = 0.11. A comparison of the ROC of APRI vs. that of FIB-4 to predict fibrosis ≥2 in patients with AST>148 IU/L is shown in S4 Fig, P = 0.19.

A comparison of the ROC of APRI vs. that of FIB-4 to predict fibrosis ≥3 in patients with AST≤37 IU/L is shown in S5 Fig, P = 0.11. A comparison of the ROC of APRI vs. that of FIB-4 to predict fibrosis ≥3 in patients with 37<AST≤74 IU/L is shown in S6 Fig, P = 0.33. A comparison of the ROC of APRI vs. that of FIB-4 to predict fibrosis ≥3 in patients with 74<AST≤148 IU/L is shown in S7 Fig, P = 0.04. A comparison of the ROC of APRI vs. that of FIB-4 to predict fibrosis ≥3 in patients with AST>148 IU/L is shown in S8 Fig, P = 0.13.

A comparison of the ROC of APRI vs. that of FIB-4 to predict fibrosis = 4 in patients with AST≤37 IU/L is shown in S9 Fig, P = 0.58. A comparison of the ROC of APRI vs. that of FIB-4 to predict fibrosis = 4 in patients with 37<AST≤74 IU/L is shown in S10 Fig, P = 0.21. A comparison of the ROC of APRI vs. that of FIB-4 to predict fibrosis = 4 in patients with 74<AST≤148 IU/L is shown in S11 Fig, P = 0.06. A comparison of the ROC of APRI vs. that of FIB-4 to predict fibrosis = 4 in patients with AST>148 IU/L is shown in S12 Fig, P = 0.14. Overall, the performance of FIB-4 in predicting different stages of fibrosis was better or equal to that of APRI irrespective of AST level.

### Use of histological necroinflammatory activity instead of AST to stratify patients

We stratified the patients into two groups: patients with minimal or mild chronic hepatitis (Histology Activity Index scores 1–8) and patients with moderate or severe chronic hepatitis (Histology Activity Index scores 9–18) [11]. The cutoff values of APRI and FIB-4 for predicting different stages of fibrosis were higher in the patients with moderate or severe chronic hepatitis compared to the patients with minimal or mild chronic hepatitis (Tables 7 and 8). A comparison of the ROC of APRI vs. that of FIB-4 to predict fibrosis ≥2 in patients with minimal-mild necroinflammation is shown in S13 Fig, P < 0.001. A comparison of the ROC of APRI vs. that of FIB-4 to predict fibrosis ≥2 in patients with moderate to severe necroinflammation is shown in S14 Fig, P = 0.001. A comparison of the ROC of APRI vs. that of FIB-4 to predict fibrosis ≥3 in patients with minimal-mild necroinflammation is shown in S15 Fig, P < 0.001. A comparison of the ROC of APRI vs. that of FIB-4 to predict fibrosis ≥3 in patients with moderate to severe necroinflammation is shown in S16 Fig, P < 0.001. A comparison of the ROC of APRI vs. that of FIB-4 to predict fibrosis = 4 in patients with minimal-mild necroinflammation is shown in S17 Fig, P < 0.001. A comparison of the ROC of APRI vs. that of FIB-4 to predict fibrosis = 4 in patients with moderate to severe necroinflammation is shown in S18 Fig, P < 0.001. Overall, the performance of FIB-4 in predicting different stages of fibrosis was better than that of APRI irrespective of necroinflammation stage.

**Table 7:** Diagnostic accuracies of APRI and FIB-4 for predicting liver fibrosis (Minimal-mild necroinflammation score 1–8, N = 1171).

Index	AUROC_*cutoff*_	Bootstrap AUROC_*cutoff*_	cutoff	sensitivity_*cutoff*_	specificity_*cutoff*_	PPV_*cutoff*_	NPV_*cutoff*_	Sensitivity + Specificity-1
To predict fibrosis ≥2								
APRI	0.63 (0.61–0.66)	0.63 (0.61–0.66)	1.0	43.6%	83.1%	66.9%	65.3%	27.9%
FIB-4	0.65(0.63–0.67)	0.65 (0.63–0.67)	1.4	91.3%	39.0%	53.9%	85.1%	34.0%
To predict fibrosis ≥3								
APRI	0.67 (0.64–0.70)	0.67 (0.64–0.70)	1.4	69.7%	64.3%	51.5%	79.6%	34.0%
FIB-4	0.71(0.69–0.74)	0.71 (0.69–0.74)	2.8	63.7%	79.2%	62.5%	80.0%	42.9%
To predict fibrosis = 4								
APRI	0.69 (0.66–0.72)	0.69 (0.66–0.72)	1.4	78.2%	60.5%	34.7%	91.2%	38.7%
FIB-4	0.73(0.70–0.76)	0.73 (0.70–0.76)	2.8	72.6%	73.9%	2.8%	90.9%	48.0%

APRI, aspartate aminotransferase (AST)- to-platelet ratio index; FIB-4, fibrosis index based on the four factors; AUROC, area under receiver operating characteristic

**Table 8:** Diagnostic accuracies of APRI and FIB-4 for predicting liver fibrosis (Moderate to severe necroinflammation score 9–18, N = 540).

Index	AUROC_*cutoff*_	Bootstrap AUROC_*cutoff*_	cutoff	sensitivity_*cutoff*_	specificity_*cutoff*_	PPV_*cutoff*_	NPV_*cutoff*_	Sensitivity + Specificity-1
To predict fibrosis ≥2								
APRI	0.65 (0.61–0.70)	0.65(0.61–0.70)	2.2	61.5%	69.3%	83.9%	40.9%	30.9%
FIB-4	0.68 (0.64–0.72)	0.68 (0.64–0.73)	3.0	67.4%	68.8%	84.8%	44.8%	36.1%
To predict fibrosis ≥3								
APRI	0.65(0.61–0.69)	0.68 (0.64–0.72)	1.4	84.3%	45.0%	70.8%	64.4%	36.5%
FIB-4	0.71 (0.67–0.75)	0.70 (0.66–0.74)	2.9	76.1%	65.1%	77.5%	63.3%	41.2%
To predict fibrosis = 4								
APRI	0.68 (0.64–0.72)	0.68 (0.64–0.72)	2.2	76.1%	59.4%	50.0%	82.3%	35.4%
FIB-4	0.70(0.66–0.74)	0.70 (0.66–0.74)	5.0	54.3%	84.9%	65.8%	77.7%	39.7%

APRI, aspartate aminotransferase (AST)- to-platelet ratio index; FIB-4, fibrosis index based on the four factors; AUROC, area under receiver operating characteristic

### Validation of the model

A bootstrap procedure was performed to determine the robustness of the model [17]. The AUROC curves from the APRI and FIB-4 bootstrap validation model were identical to the full-sample estimation, with the exception of only a slight change in the 95% confidence interval derived from the bootstrap model, which validated the high robustness of the proposed ROC curves.

### Performance of compound surrogates for advanced fibrosis in male, elderly, or obese patients

To assess the performance of compound surrogates for advanced fibrosis in patients who may have a peculiar distribution of liver fibrosis stages, further analysis was conducted on three subgroups of patients: (1) male patients, who are expected to have a higher prevalence of advanced fibrosis stages; (2) elderly patients (age ≥65 years), who are also expected to have a higher prevalence of advanced fibrosis stages; and (3) obese patients, who are also expected to have a higher prevalence of advanced fibrosis stages. Overall, 913 (53.2%) patients were male, 220 (12.8%) patients were over 65 years of age, 493 (28.7%) patients were overweight (BMI 24–27 kg/m^2^), and 355 (20.1%) patients were obese (BMI> 27 kg/m^2^) [18]. Comparisons of the demographic, laboratory, and histological features of the patients according to gender, age, and BMI are summarized in S1–S3 Tables. When compared with the female patients, the male patients had a higher prevalence of advanced fibrosis stages (47.6% vs. 39.7%, P = 0.027), an older mean age (54.6 vs. 50.9 years, P<0.001), a higher prevalence of thrombocytopenia (43.7% vs. 36%, P = 0.001), and a lower median ALT level (126 IU/L vs. 143 IU/L, P<0.001). When compared with the non-elderly patients, the elderly patients had a higher prevalence of advanced fibrosis stages (61.8% vs. 40.7%, P < 0.0001), a higher prevalence of thrombocytopenia (59.1% vs. 36.8%, P<0.001), and a higher median AST level (103.5 IU/L versus 89 IU/L, P = 0.002). When compared with normal weight patients, the patients who were overweight or obese had a higher prevalence of advanced fibrosis stages (48.2% in obese patients, 48.8% in overweight patients, and 37.3% in normal weight patients, P<0.001).

The AUROCs of FIB-4 for predicting ≥F3 and cirrhosis were >0.7 in male and female patients, while the AUROCs of FIB-4 for predicting ≥F2 were 0.71 in male patients and 0.69 in female patients. The AUROCs of APRI for predicting ≥F2 and ≥F3 were <0.7 in male and female patients, while the AUROCs of APRI for predicting cirrhosis were both 0.70 in male and female patients (S4 and S5 Tables). Overall, the performances of APRI and FIB-4 were not different between genders, except that the performance of FIB-4 in predicting ≥F2 was better in male patients.

The AUROCs of APRI to predict ≥F2 and ≥F3 were <0.70 in elderly and non-elderly patients. The AUROCs of APRI to predict cirrhosis were 0.60 in elderly patients and 0.72 in non-elderly patients. The AUROCs of FIB-4 to predict different stages of fibrosis were < 0.7 in elderly patients and >0.7 in non-elderly patients (S6 and S7 Tables). Overall, the performances of APRI and FIB-4 were better in non-elderly patients.

The AUROCs of APRI to predict different stages of liver fibrosis were >0.70 in obese patients and <0.70 in normal weight and overweight patients. The AUROCs of FIB-4 to predict different stages of liver fibrosis were >0.7 irrespective of BMI (S8 and S9 Tables). Overall, the performance of APRI was better in obese patients. However, the performance of FIB-4 was not affected by BMI.

### Correlations between APRI, FIB-4, and clinical variables

APRI and FIB-4 were positively correlated with age, AST, ALT level, international normalized ratio (INR), necroinflammation score, and fibrosis score, and were negatively correlated with albumin level and platelet count (S10 Table).

### Performance of AST/ALT ratio ≥1 in distinguishing cirrhotic from non-cirrhotic patients

AST/ALT ratios ≥1 had 91.5% specificity in distinguishing cirrhotic from non-cirrhotic patients; the AUROC was 0.57, the sensitivity was 21.6%, the PPV was 46.3%, and the NPV was 77.4% in all patients. In the subgroup analysis stratified by AST level, the specificity of AST/ALT ratios ≥1 in distinguishing cirrhotic from non-cirrhotic patients was 77.8% in patients with AST<37 IU/L, 94.5% in patients with 37≤AST<74 IU/L, 94.3% in patients with 74<AST≤148 IU/L, and 87.1% in patients with AST>148IU/L (S11 Table).

## Discussion

While liver biopsies are frequently used for diagnostic purposes, the procedure also has various limitations, including being invasive and costly, as well as potentially resulting in sampling errors and inter- and intraobserver discrepancies in assessing hepatic fibrosis [19–21]. Relatedly, the procedure does not play a role in the staging of patients with HCV today. Rather, according to the recommendations of the EASL guidelines, fibrosis staging must be assessed by non-invasive methods initially [22].

Transient elastography (TE) can be considered the non-invasive standard for the measurement of hepatic fibrosis [7]. TE is well validated in CHC patients. However, TE also has limitations, mainly the reduced accuracy of the test in obese patients and patients with hepatic inflammation [23, 24].

Because of the aforementioned limitations of liver biopsy and TE, several compound surrogates for advanced fibrosis have been proposed and validated in CHC patients [7], with the greatest amount of attention having been centered on the APRI and the FIB-4. These compound surrogates for advanced fibrosis have been of greater interest to clinicians because they are simple to calculate and readily available during daily clinical practice.

In a meta-analysis of 40 studies, investigators concluded that an APRI score greater than 1.0 had a sensitivity of 76% and a specificity of 72% for predicting cirrhosis. In addition, they concluded that an APRI score greater than 0.7 had a sensitivity of 77% and a specificity of 72% for predicting significant hepatic fibrosis [25]. In the present study, the optimal cutoff values of APRI for the diagnosis of ≥ F2 (significant fibrosis) were found to be 0.5 in patients with AST≤37 IU/L, 1.1 in patients with 37<AST≤74 IU/L, 1.9 in patients with 74<AST≤148 IU/L, 3.4 in patients with AST>148 IU/L, and 1.4 in all patients. The optimal cutoff values of APRI for the diagnosis of cirrhosis were found to be 0.6 in patients with AST≤37 IU/L, 1.1 in patients with 37<AST≤74 IU/L, 2.2 in patients with 74<AST≤148 IU/L, 3.4 in patients with AST>148 IU/L, and 2.2 in all patients.

According to a previous study, FIB-4 >3.25 would have a 97% specificity and a positive predictive value of 65% for advanced fibrosis [12]. In this study, meanwhile, the optimal cutoff values of FIB-4 for the diagnosis of ≥F3 (advanced fibrosis) were found to be 1.4 in patients with AST≤37 IU/L, 2.2 in patients with 37<AST≤74 IU/L, 3.2 in patients with 74<AST≤148 IU/L, 5.2 in patients with AST>148 IU/L, and 2.9 in all patients.

Furthermore, the results of this study indicated that the optimal cutoff values of APRI and FIB-4 for predicting liver fibrosis were obviously increased as the AST levels increased. We also found that, in comparison to those with AST ≤148 IU/L, those with AST> 148 IU/L had higher necroinflammation scores and included higher proportions of patients who had cirrhosis. The association of elevated AST with the progression of liver fibrosis could be explained as follows: the progression of liver fibrosis may reduce the clearance of AST [26], leading to increased serum AST levels. Moreover, advanced liver disease may be associated with mitochondrial injury, resulting in the marked release of AST [27].

In this large observational real-world cohort of CHC patients, the AUROC of FIB-4 for predicting advanced fibrosis (i.e.≥F3) was 0.73 in all patients. Compared to other studies [28], the accuracy of FIB-4 for predicting liver fibrosis was rather low in our study. The spectrum effect (when the prevalence of each fibrosis stage varies widely between studies) is a major cause of discrepancy in the accuracy of FIB-4 for predicting liver fibrosis between studies [28].

To the best of our knowledge, this is the first analysis of these compound surrogates for advanced fibrosis stratified by AST levels in CHC patients. The use of APRI and FIB-4 has been found to entail a risk of overestimating the fibrosis stage due to the impact of necroinflammatory activity on transaminases [7]. In this study, histological necroinflammatory activity was used instead of AST to stratify CHC patients. The cutoff values of APRI and FIB-4 to predict different stages of fibrosis were found to be higher in patients with moderate or severe chronic hepatitis than in patients with minimal or mild chronic hepatitis.

Our study has also shown that the performance of APRI and FIB-4 for liver fibrosis may be distinct in special subgroups of patients who are expected to have a higher prevalence of advanced fibrosis stages, such as male patients, elderly patients, and obese patients, in contrast to in normal AST patients who are expected to have a lower prevalence of advanced fibrosis stages. Overall, the performances APRI and FIB-4 were not different between genders, except that the performance of FIB-4 in predicting ≥F2 was better in male patients. The performances of APRI and FIB-4 were also better in non-elderly patients, while the performance of APRI was better in obese patients. However, the performance of FIB-4 was not affected by BMI. The performances of APRI and FIB-4 were not different in patients with normal AST compared to patients with elevated AST. Few studies have specifically investigated the performance of APRI and FIB-4 in such patients.

In this study, we found that APRI and FIB-4 were correlated with age, AST level, ALT level, fibrosis score, and platelet count, as expected. Furthermore, APRI and FIB-4 were correlated with INR, albumin level, and necroinflammation score. The positive correlations of APRI and FIB-4 with necroinflammation score also supported our hypothesis that the use of APRI and FIB-4 entails a risk of overestimating the fibrosis stage due to the impact of necroinflammatory activity on transaminases [7].

The AST/ALT ratio is another commonly used compound surrogate for cirrhosis, with transaminases being included in this formula. A previous study reported that AST/ALT ratios ≥1 had 100% specificity in distinguishing cirrhotic from non-cirrhotic CHC patients [29]. In our study, AST/ALT ratios ≥1 had 91.5% specificity in distinguishing cirrhotic from non-cirrhotic patients in all patients, a finding which was compatible with the aforementioned previous study [29]. However, in the subgroup analysis stratified by AST level, the specificity was >90% in patients with 37≤AST<74 IU/L and patients with 74≤AST<148 IU/L, but the specificity was < 90% in patients with AST <37IU/L and patients with AST>148 IU/L. Therefore, clinicians should be careful about using AST/ALT ratios≥1 to rule in cirrhosis patients in the cases of those with normal AST levels and those with marked AST elevation.

The present study has several strengths. We enrolled a large population of CHC patients with a wide spectrum of liver disease, ranging from the absence of fibrosis to compensated cirrhosis. At the same time, we acknowledge that there are limitations to our analysis. First, this was a retrospective study, and our patients were enrolled from a single referral center; thus, selection bias may have occurred. Second, our study did not involve a central pathologist for the interpretation of liver histology. Thus, there were interobserver discrepancies in the assessments of hepatic fibrosis. Third, although the bootstrap method was undertaken for internal validation [17], a more rigorous validation with an independent external cohort is still needed to confirm the optimal cutoff values of the APRI and FIB-4 in predicting liver fibrosis in patients with CHC stratified by AST levels. Fourth, because this study was an observational study, the data collected from the medical records were solely based on routine clinical care and thus representative of the “real world” clinical setting. As such, the blood tests necessary for imputation of the serum fibrosis markers were not necessarily collected on the same date as the liver biopsy was conducted. Nonetheless, the goal of our study was to evaluate the capability of the compound surrogates for advanced fibrosis, imputed from blood tests collected during the course of routine care and within 3 months of liver biopsy, to predict liver fibrosis in a real-world setting.

In conclusion, the diagnostic accuracy of FIB-4 for predicting liver fibrosis was found to be equal to or better than that of APRI when stratified by AST levels in this study. However, APRI is recommended as the preferred noninvasive test for assessing the presence of cirrhosis in resource-limited settings according to the Asian Pacific Association for the Study of the Liver (APASL) guidelines [30]. Relatedly, this study provides optimal cutoff values stratified by AST levels for both serum markers, which could be more feasible compared with the single cutoff values proposed in previous studies [12, 25, 28].

## Supporting information

S1 Fig A comparison of the ROC of APRI vs. that of FIB-4 to predict fibrosis ≥2 in patients with AST≤37 IU/L. (TIF)

S2 Fig A comparison of the ROC of APRI vs. that of FIB-4 to predict fibrosis ≥2 in patients with 37<AST≤74 IU/L. (TIF)

S3 Fig A comparison of the ROC of APRI vs. that of FIB-4 to predict fibrosis ≥2 in patients with 74<AST≤148 IU/L. (TIF)

S4 Fig A comparison of the ROC of APRI vs. that of FIB-4 to predict fibrosis ≥2 in patients with AST>148 IU/L. (TIF)

S5 Fig A comparison of the ROC of APRI vs. that of FIB-4 to predict fibrosis ≥3 in patients with AST≤37 IU/L. (TIF)

S6 Fig A comparison of the ROC of APRI vs. that of FIB-4 to predict fibrosis ≥3 in patients with 37<AST≤74 IU/L. (TIF)

S7 Fig A comparison of the ROC of APRI vs. that of FIB-4 to predict fibrosis ≥3 in patients with 74<AST≤148 IU/L. (TIF)

S8 Fig A comparison of the ROC of APRI vs. that of FIB-4 to predict fibrosis ≥3 in patients with AST>148 IU/L. (TIF)

S9 Fig A comparison of the ROC of APRI vs. that of FIB-4 to predict fibrosis = 4 in patients with AST≤37 IU/L. (TIF)

S10 Fig A comparison of the ROC of APRI vs. that of FIB-4 to predict fibrosis = 4 in patients with 37<AST≤74 IU/L. (TIF)

S11 Fig A comparison of the ROC of APRI vs. that of FIB-4 to predict fibrosis = 4 in patients with 74<AST≤148 IU/L. (TIF)

S12 Fig A comparison of the ROC of APRI vs. that of FIB-4 to predict fibrosis = 4 in patients with AST>148 IU/L. (TIF)

S13 Fig A comparison of the ROC of APRI vs. that of FIB-4 to predict fibrosis ≥2 in patients with minimal-mild necroinflammation. (TIF)

S14 Fig A comparison of the ROC of APRI vs. that of FIB-4 to predict fibrosis ≥2 in patients with moderate to severe necroinflammation. (TIF)

S15 Fig A comparison of the ROC of APRI vs. that of FIB-4 to predict fibrosis ≥3 in patients with minimal-mild necroinflammation. (TIF)

S16 Fig A comparison of the ROC of APRI vs. that of FIB-4 to predict fibrosis ≥3 in patients with moderate to severe necroinflammation. (TIF)

S17 Fig A comparison of the ROC of APRI vs. that of FIB-4 to predict fibrosis = 4 in patients with minimal-mild necroinflammation. (TIF)

S18 Fig A comparison of the ROC of APRI vs. that of FIB-4 to predict fibrosis = 4 in patients with moderate to severe necroinflammation. (TIF)

S1 Table Comparison of demographic, laboratory and histological characteristics in male versus female patients. (DOCX)

S2 Table Comparison of demographic, laboratory and histological characteristics in patients with age ≥65 years versus age<65 years. (DOCX)

S3 Table Comparison of demographic, laboratory and histological characteristics in patients with normal weight, overweight and obese. (DOCX)

S4 Table Comparison of diagnostic accuracies of APRI for predicting liver fibrosis in male versus female patients. (DOCX)

S5 Table Comparison of diagnostic accuracies of FIB-4 for predicting liver fibrosis in male versus female patients. (DOCX)

S6 Table Comparison of diagnostic accuracies of APRI for predicting liver fibrosis in elderly (age ≥65 years) and non-elderly (age <65 years) patients. (DOCX)

S7 Table Comparison of diagnostic accuracies of FIB-4 for predicting liver fibrosis in elderly (age ≥65 years) and non-elderly (age <65 years) patients. (DOCX)

S8 Table Comparison of diagnostic accuracies of APRI for predicting liver fibrosis in noraml weight, overweight and obese patients. (DOCX)

S9 Table Comparison of diagnostic accuracies of FIB-4 for predicting liver fibrosis in noraml weight, overweight and obese patients. (DOCX)

S10 Table Correlation between APRI, FIB-4 and clinical variables (N = 1716). (DOCX)

S11 Table Performance of AST/ALT ratio ≥1 in distinguishing cirrhotic from non-cirrhotic patients. (DOCX)

S1 Data Raw data.xlsx: Including age, gender, platelet count, body mass index, biochemistry and histology. (XLSX)
