# Supplementary figures and images for: APRI and FIB-4 in the evaluation of liver fibrosis in chronic hepatitis C patients stratified by AST level
Source: PLoS One. 2018 Jun 28;13(6):e0199760. doi: 10.1371/journal.pone.0199760 (PMC6023204; doi:10.1371/journal.pone.0199760)

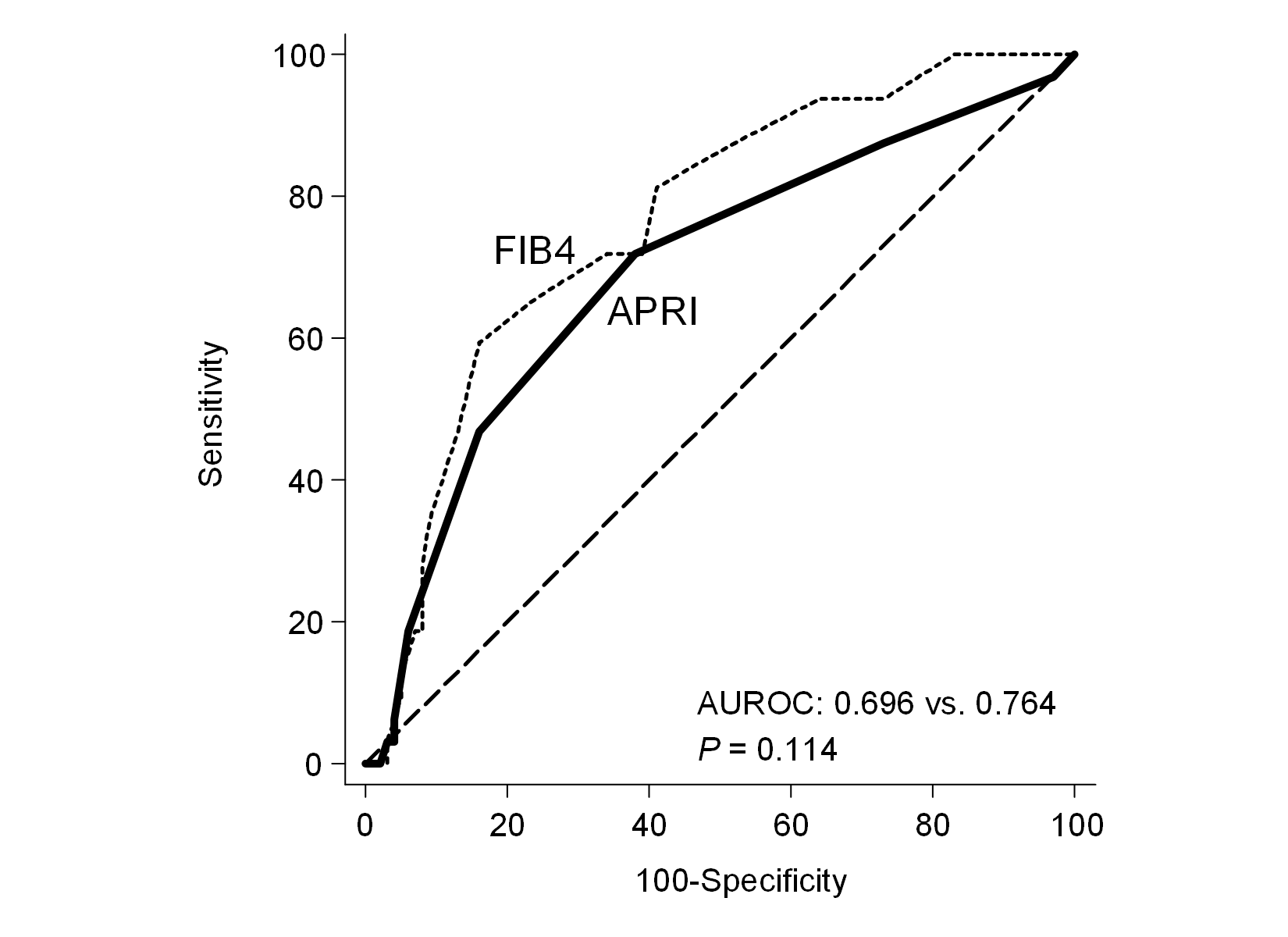

Supplement: S1 Fig — (TIF) [file pone.0199760.s001.tif]

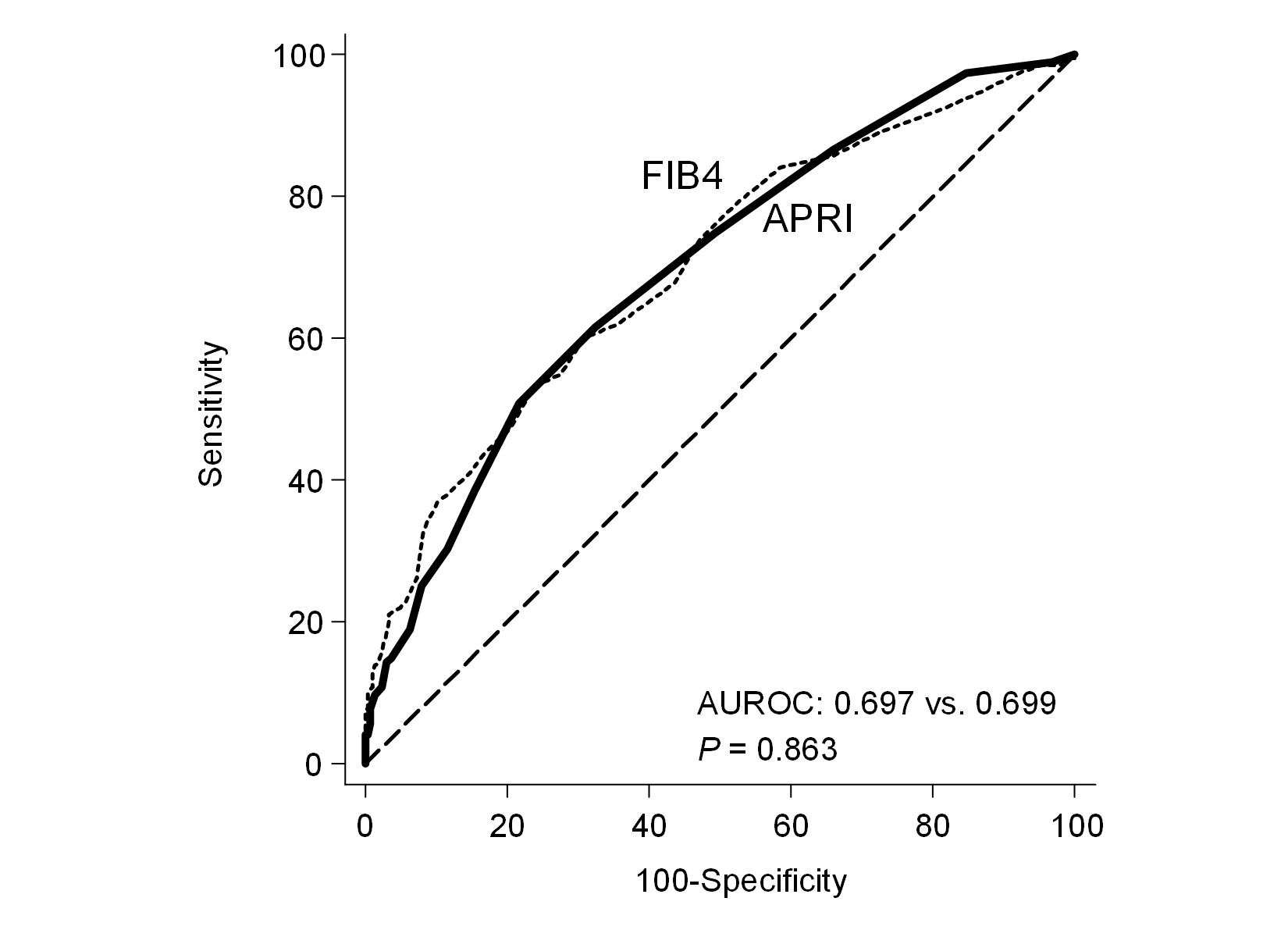

Supplement: S2 Fig — (TIF) [file pone.0199760.s002.tif]

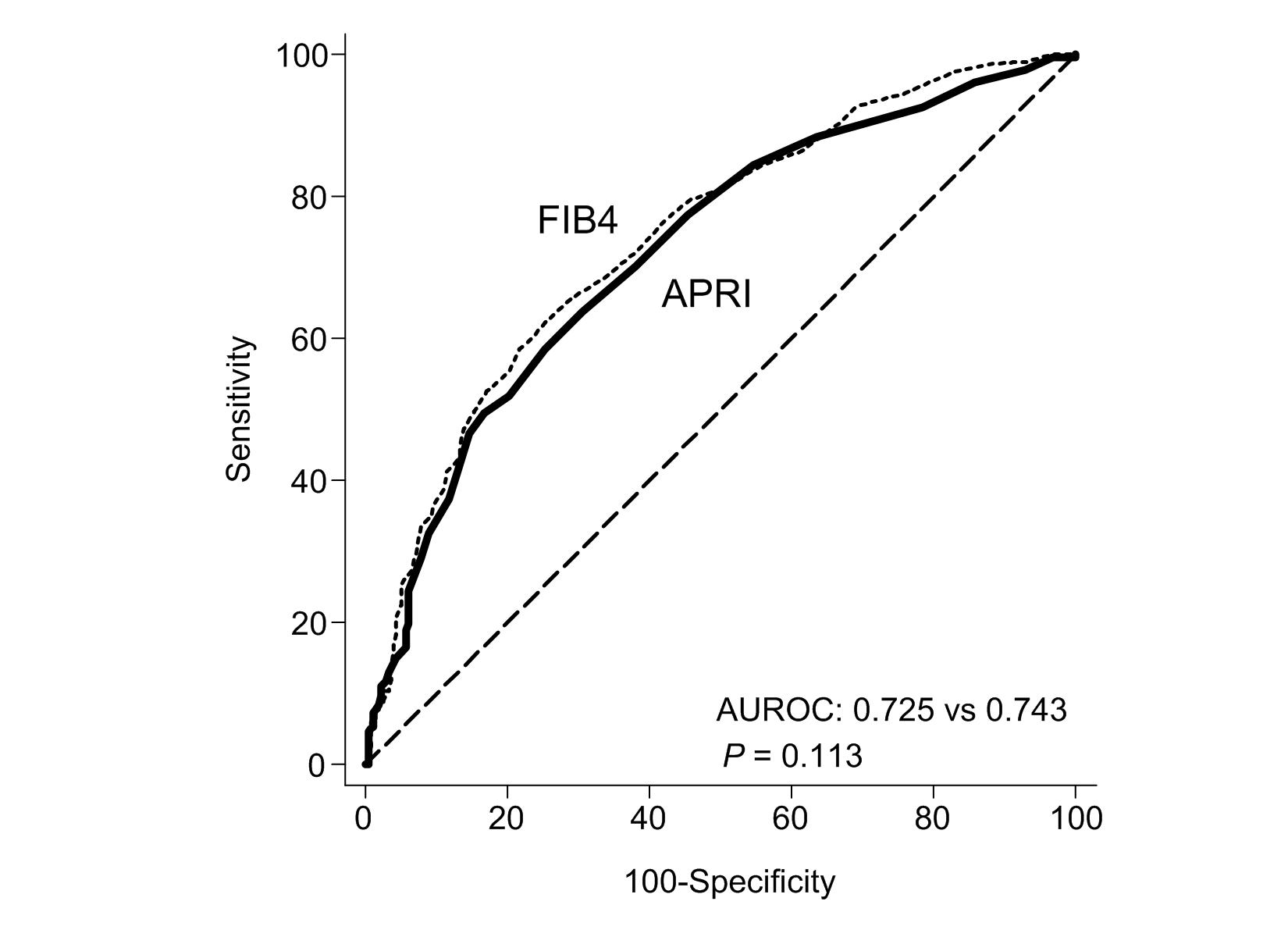

Supplement: S3 Fig — (TIF) [file pone.0199760.s003.tif]

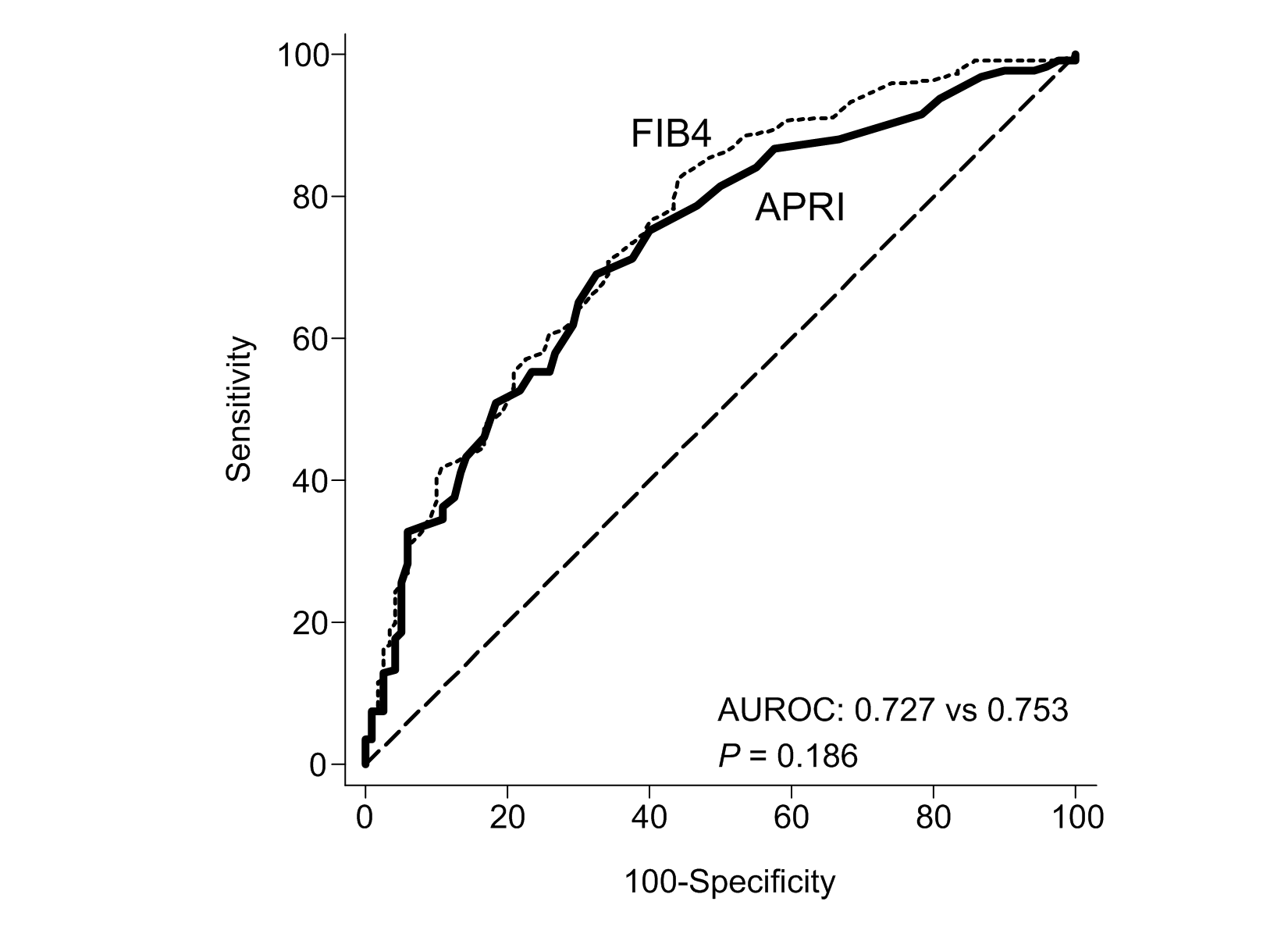

Supplement: S4 Fig — (TIF) [file pone.0199760.s004.tif]

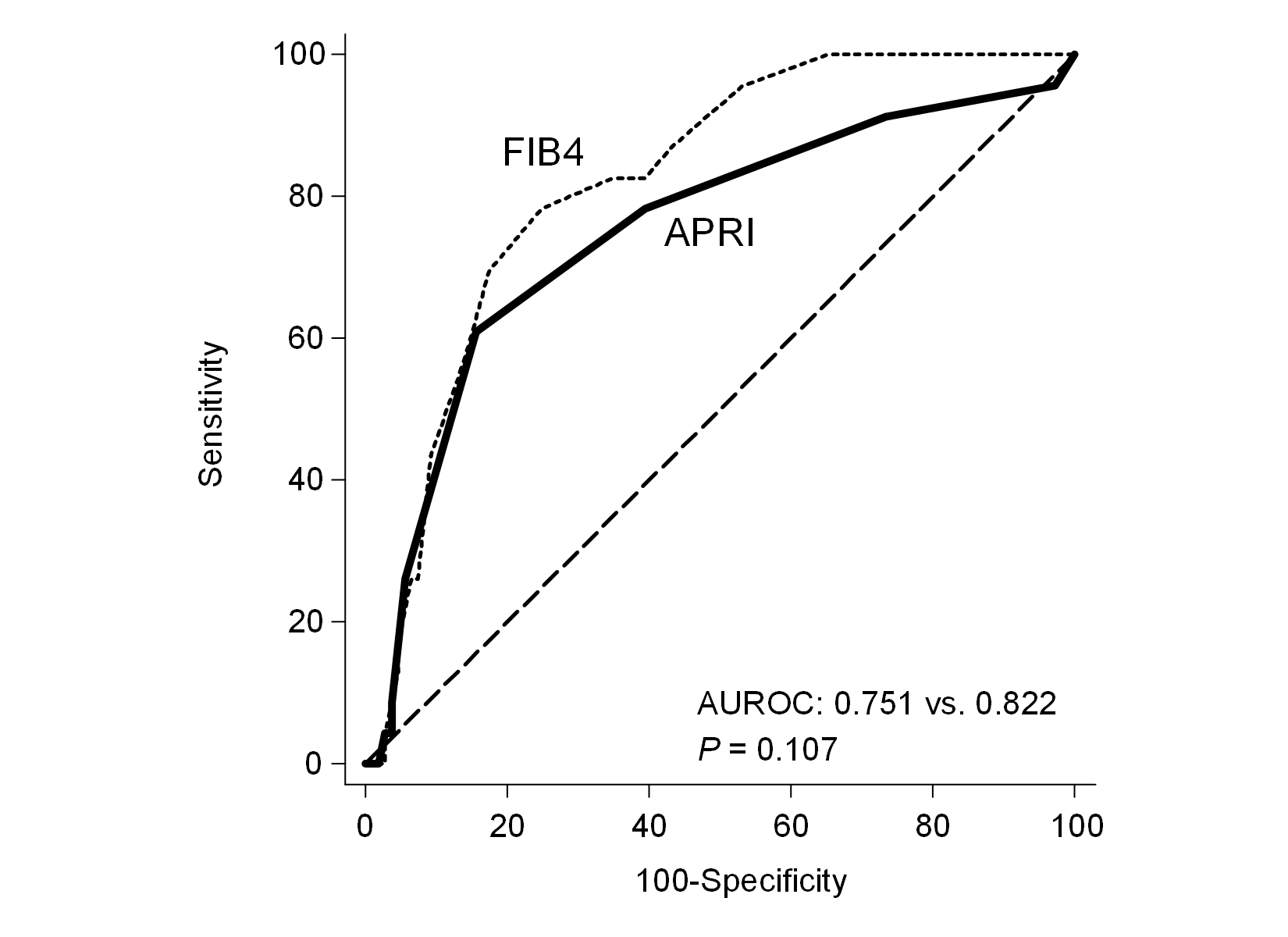

Supplement: S5 Fig — (TIF) [file pone.0199760.s005.tif]

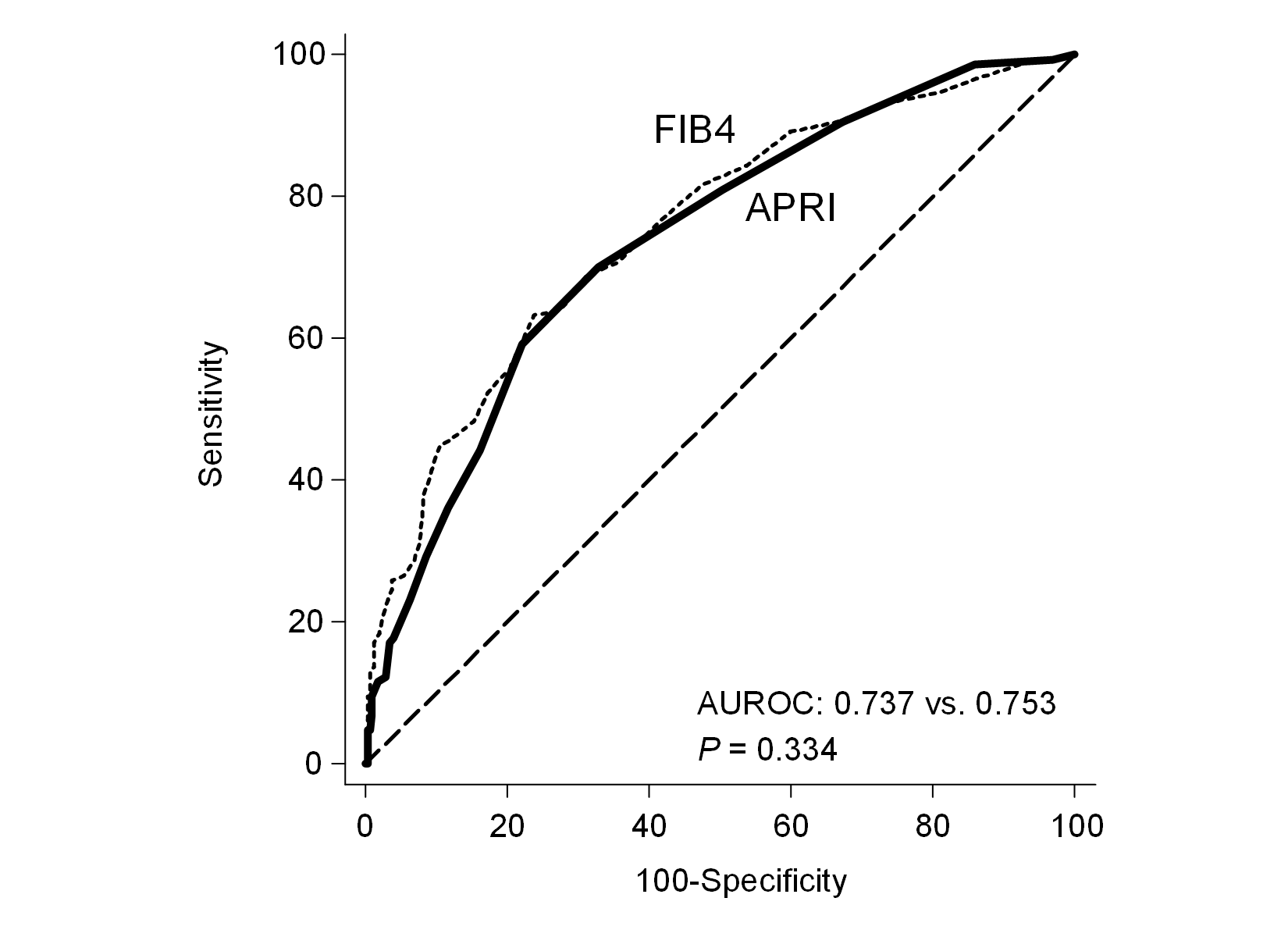

Supplement: S6 Fig — (TIF) [file pone.0199760.s006.tif]

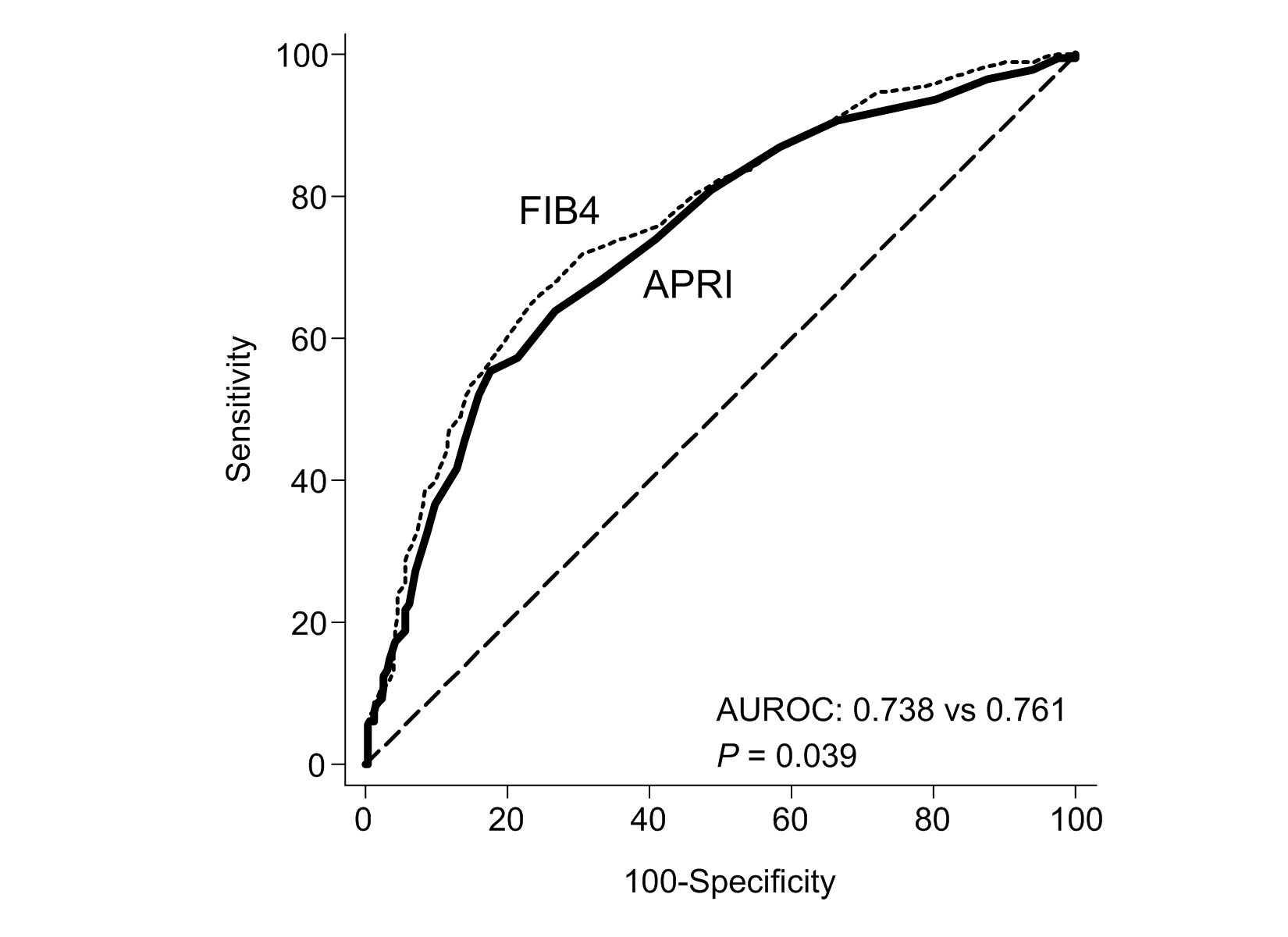

Supplement: S7 Fig — (TIF) [file pone.0199760.s007.tif]

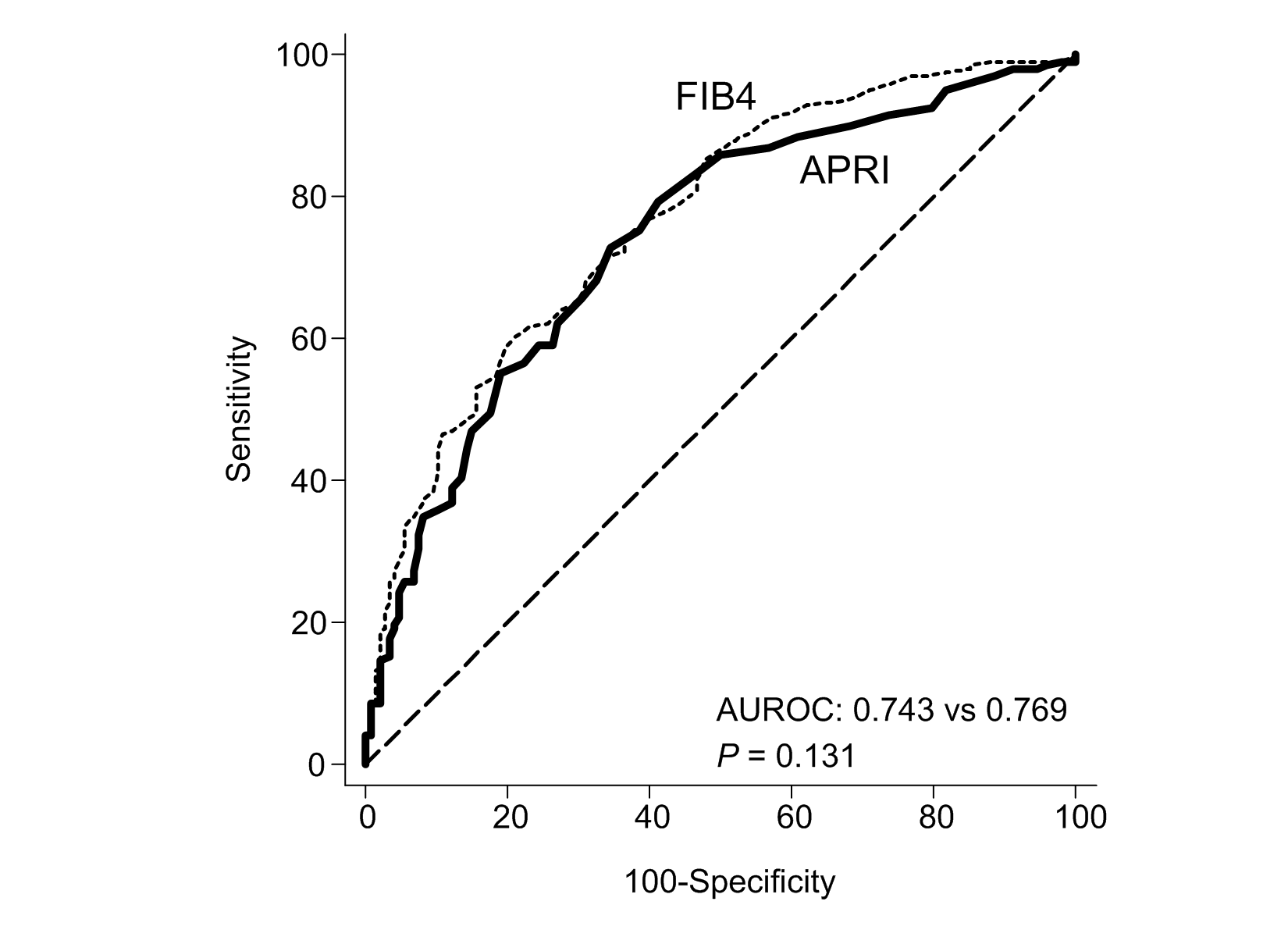

Supplement: S8 Fig — (TIF) [file pone.0199760.s008.tif]

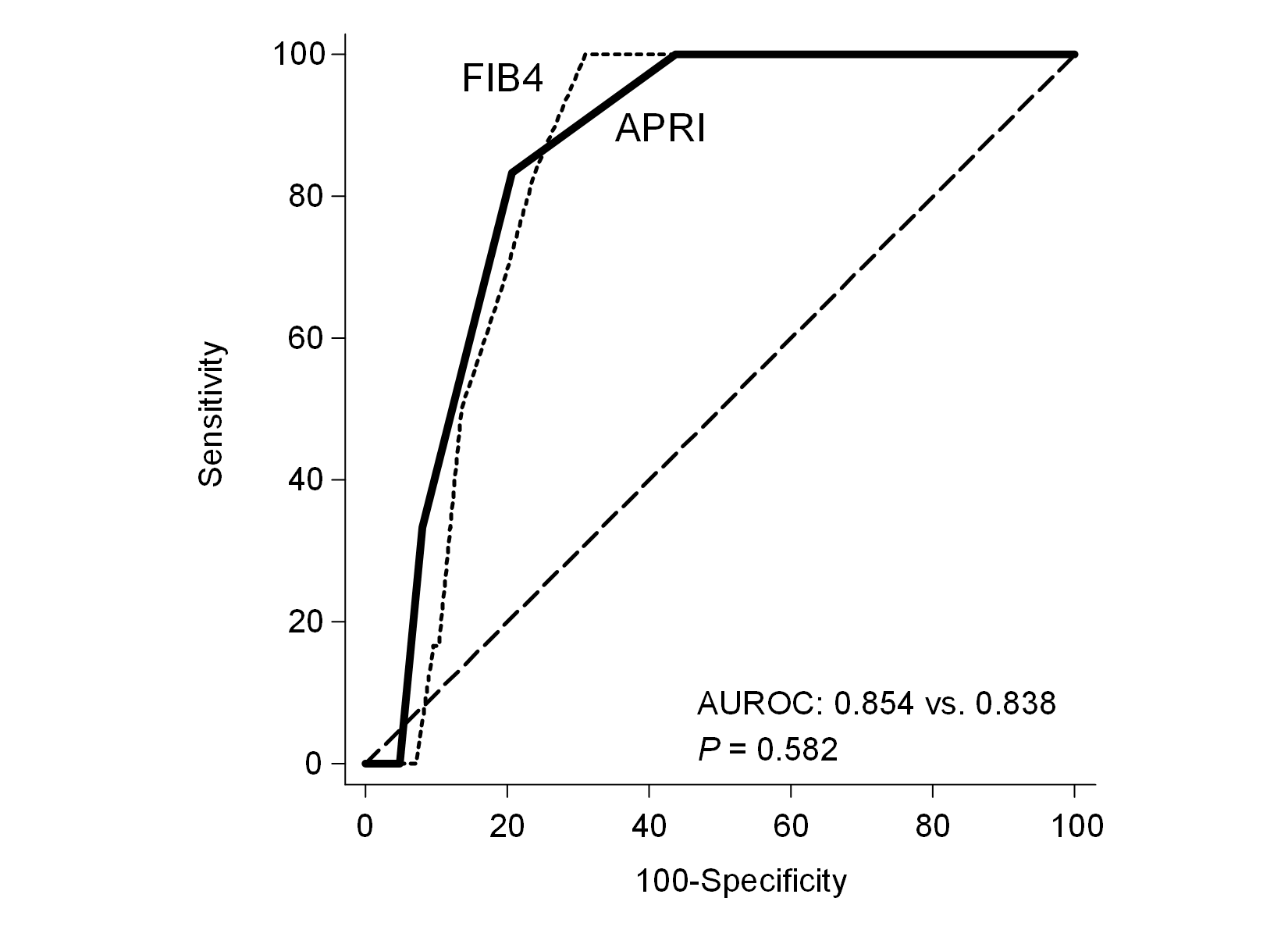

Supplement: S9 Fig — (TIF) [file pone.0199760.s009.tif]

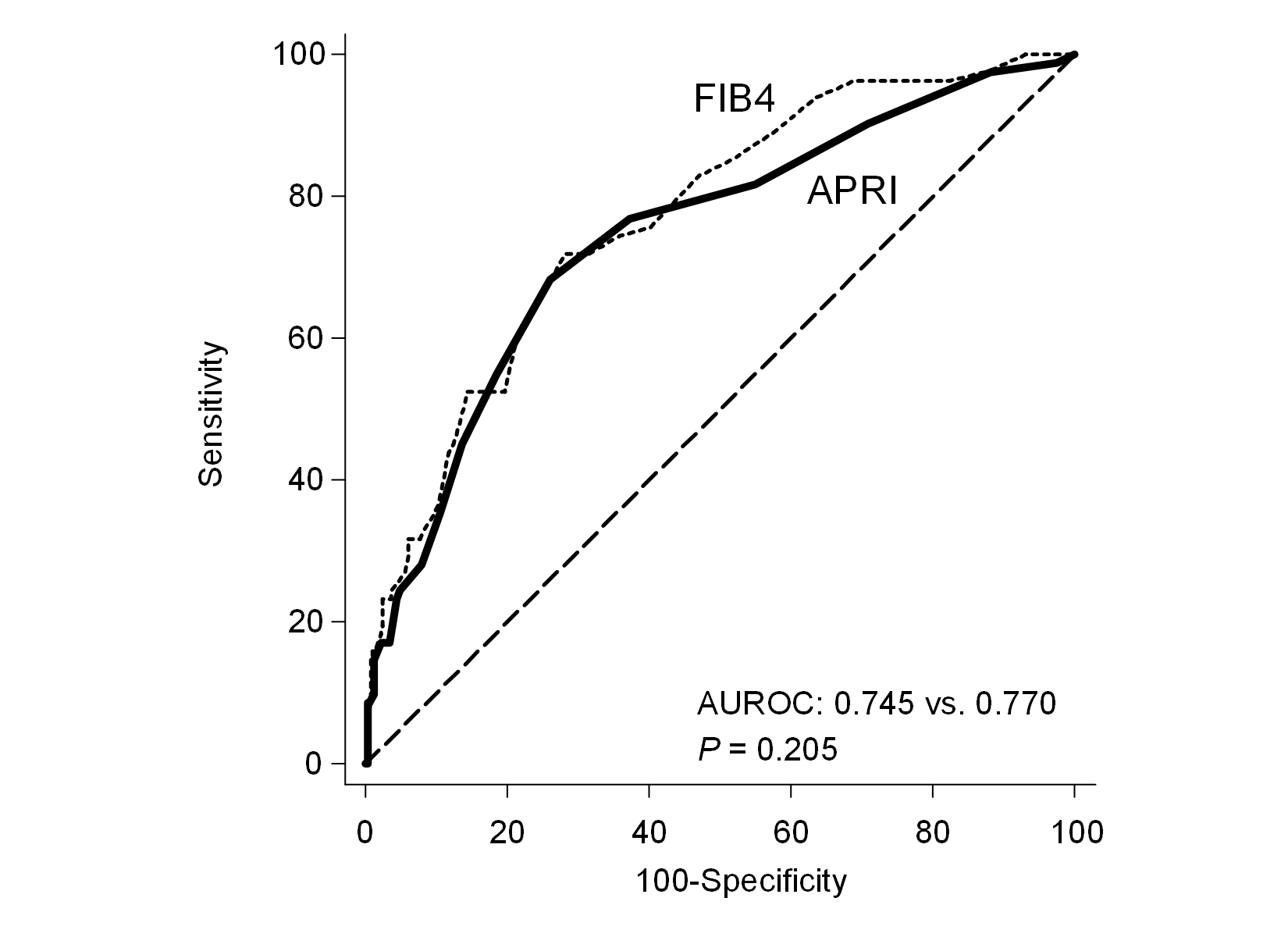

Supplement: S10 Fig — (TIF) [file pone.0199760.s010.tif]

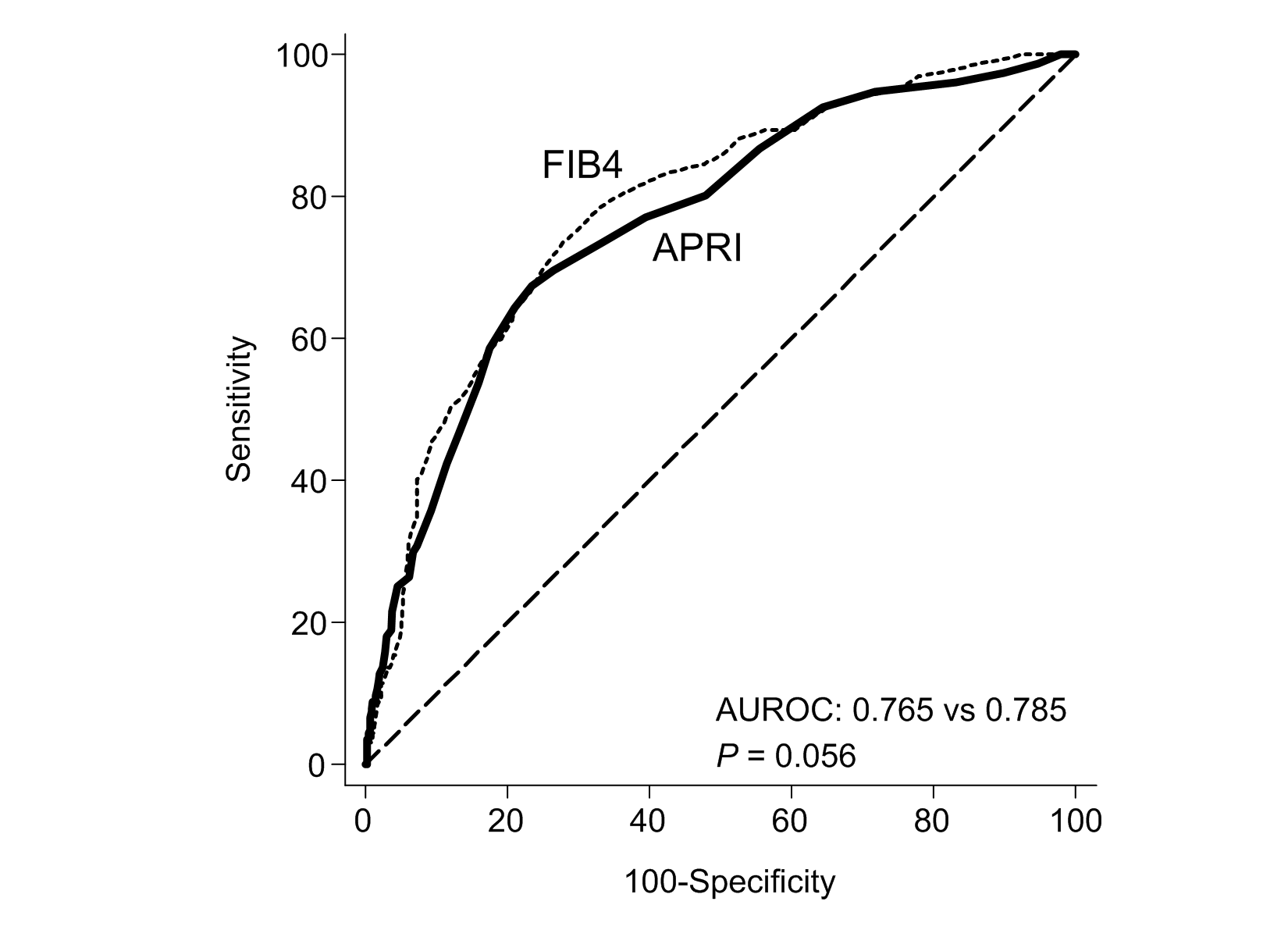

Supplement: S11 Fig — (TIF) [file pone.0199760.s011.tif]

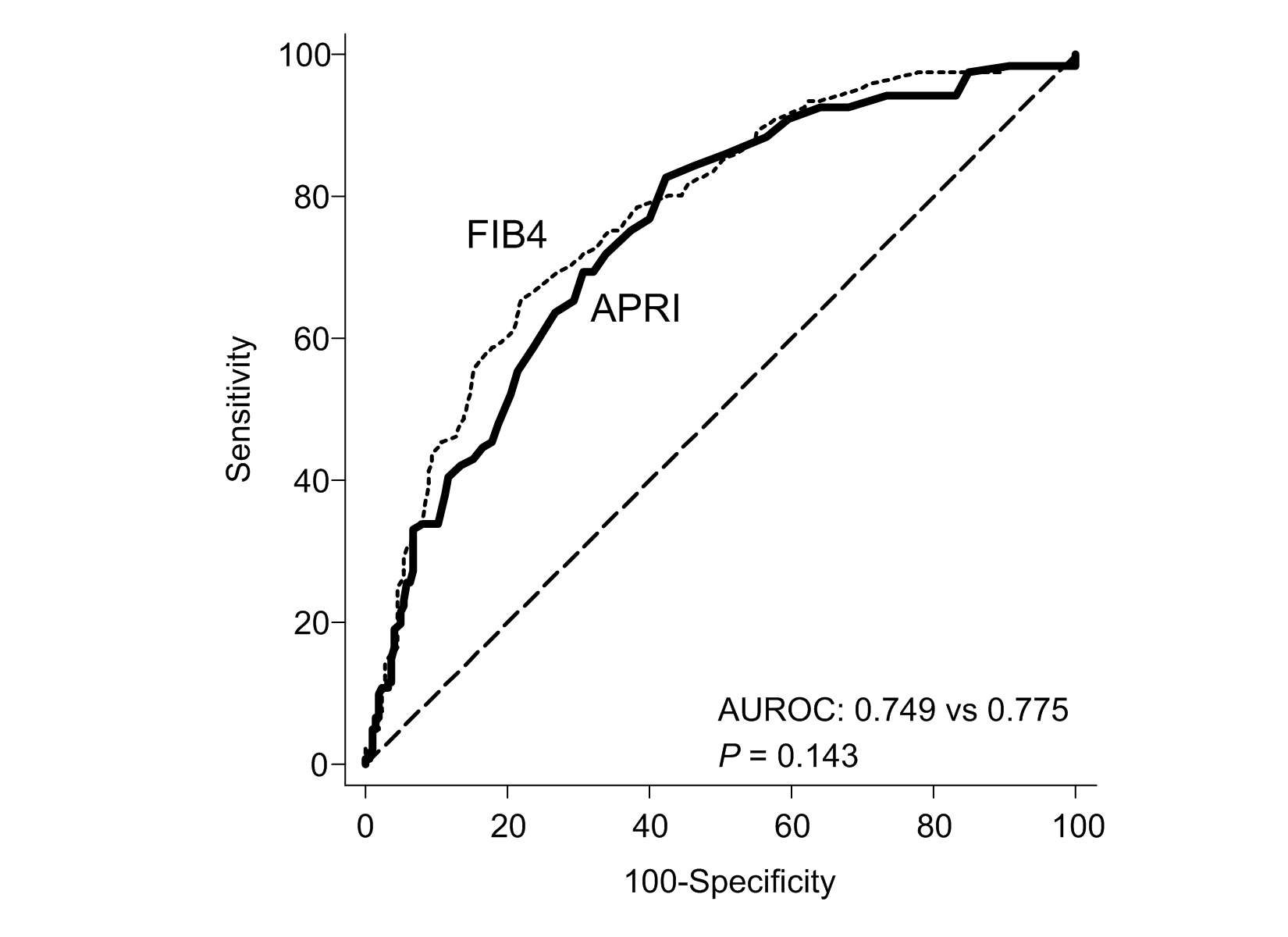

Supplement: S12 Fig — (TIF) [file pone.0199760.s012.tif]

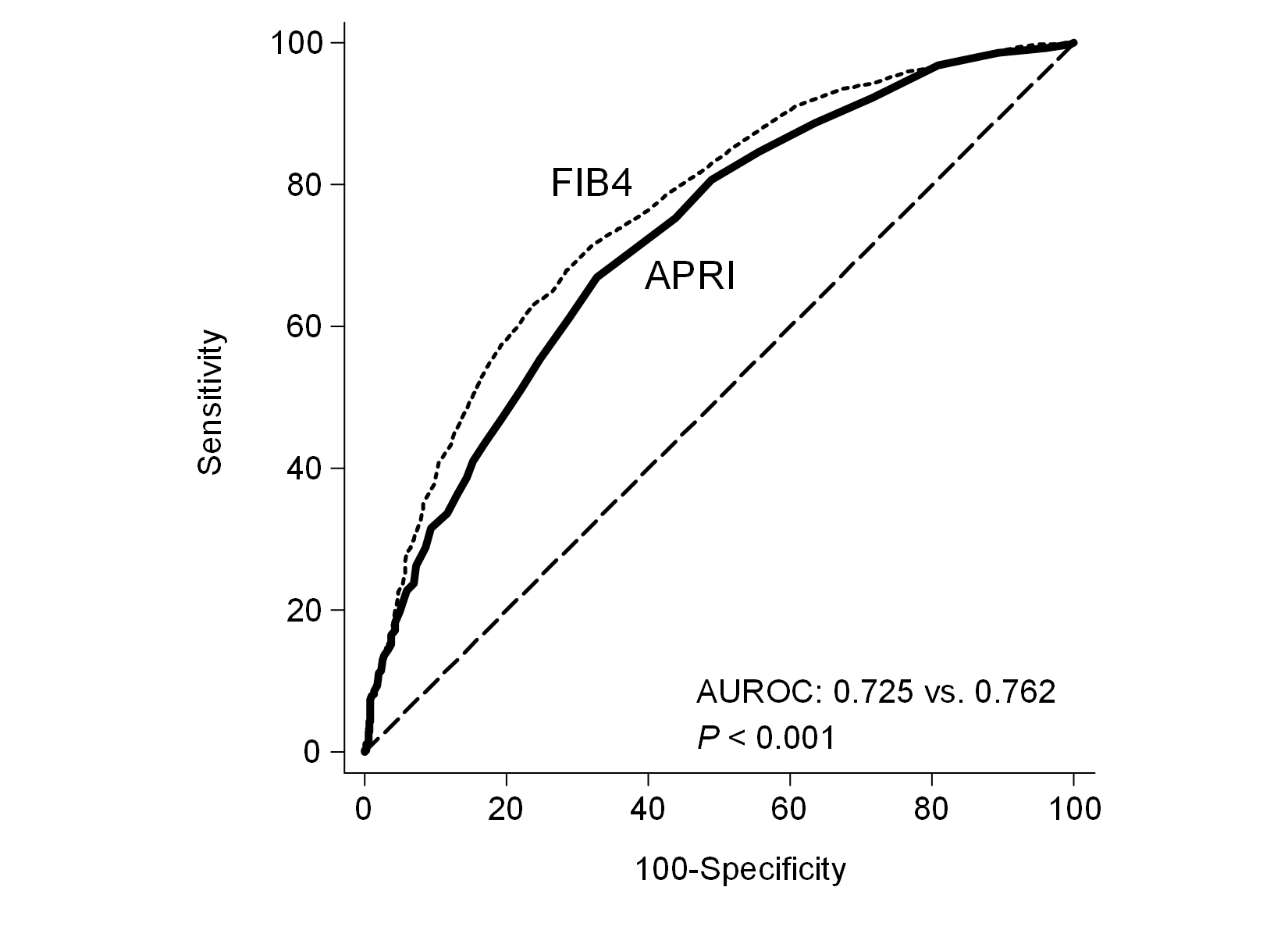

Supplement: S13 Fig — (TIF) [file pone.0199760.s013.tif]

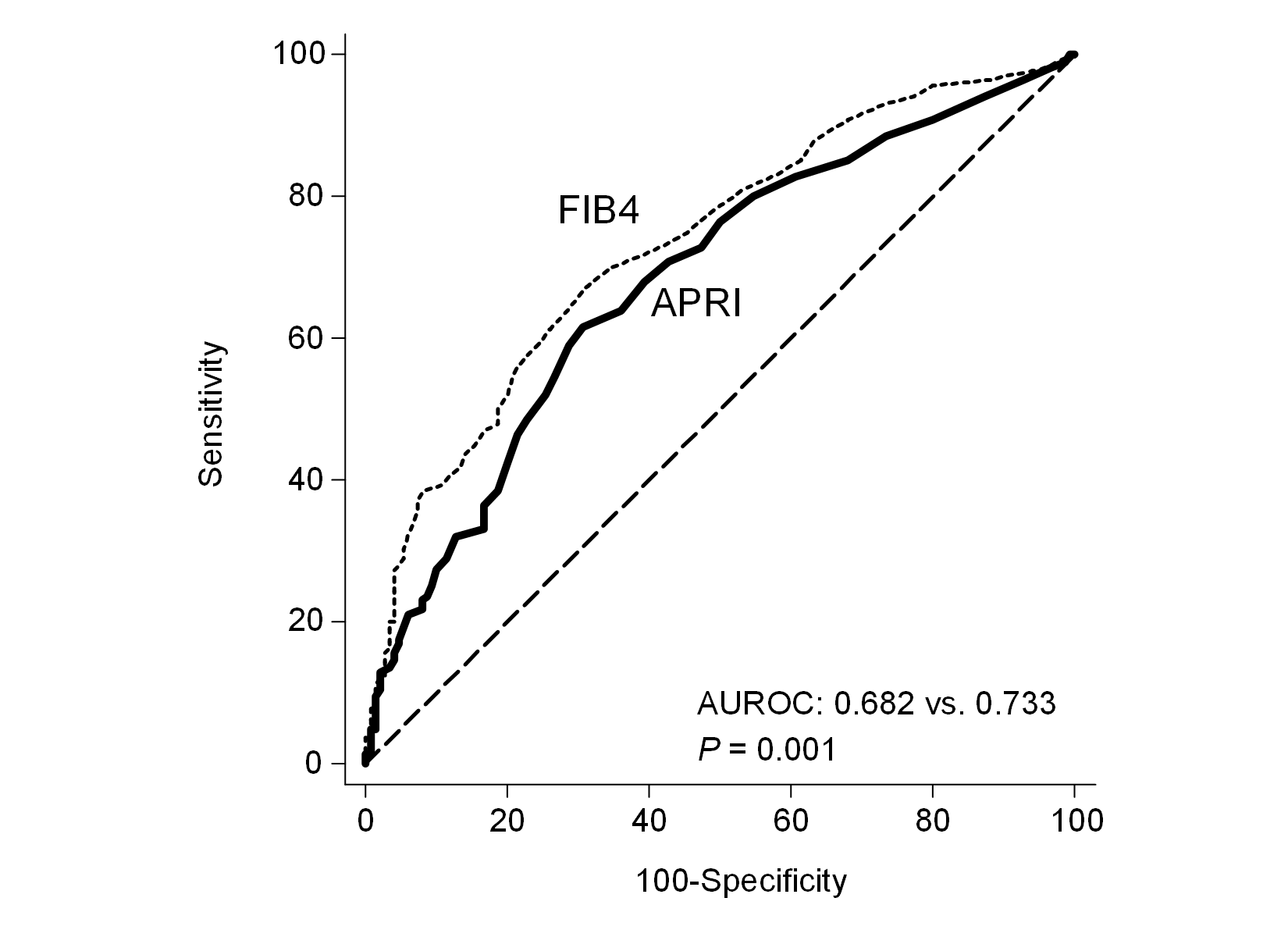

Supplement: S14 Fig — (TIF) [file pone.0199760.s014.tif]

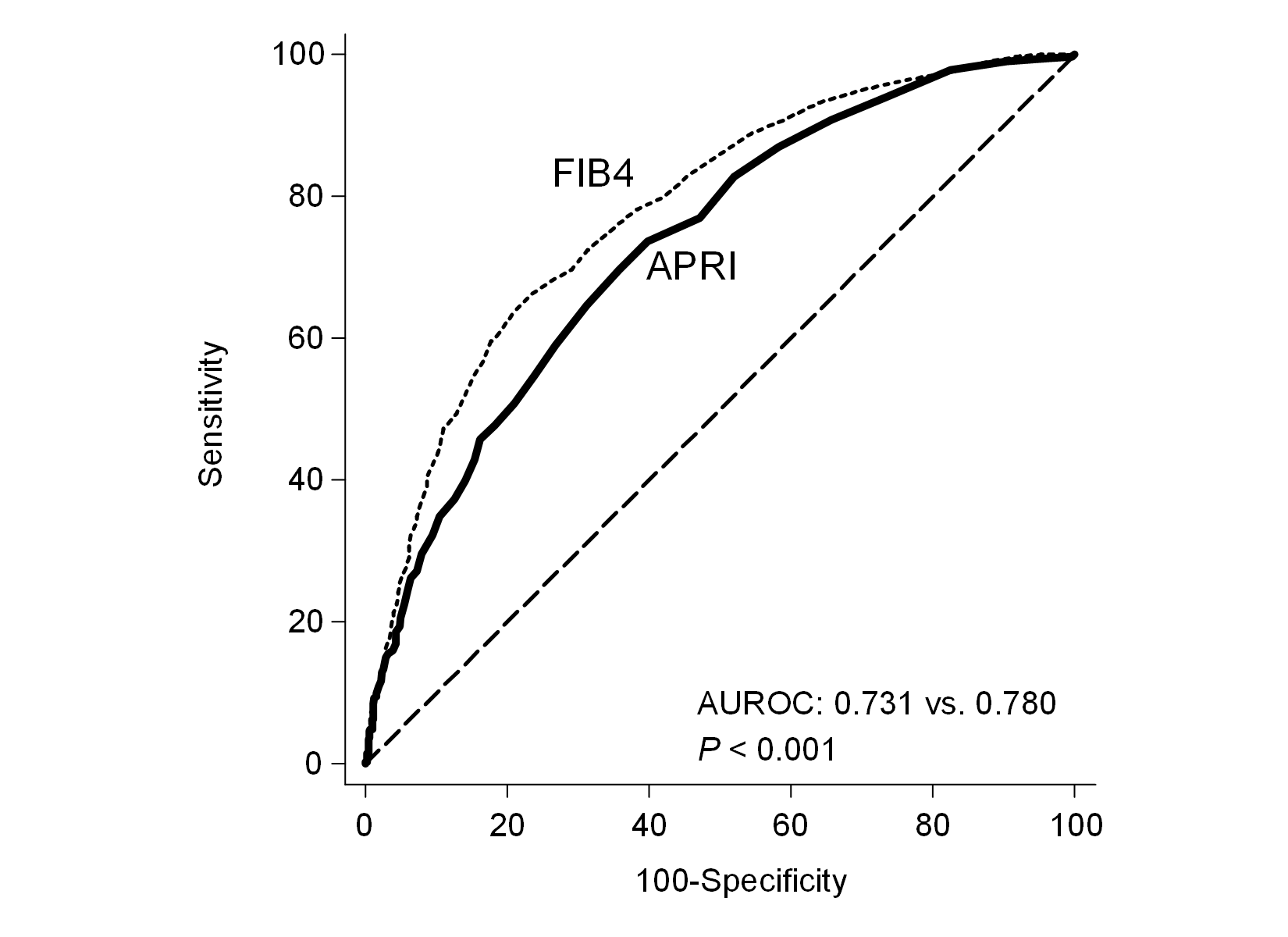

Supplement: S15 Fig — (TIF) [file pone.0199760.s015.tif]

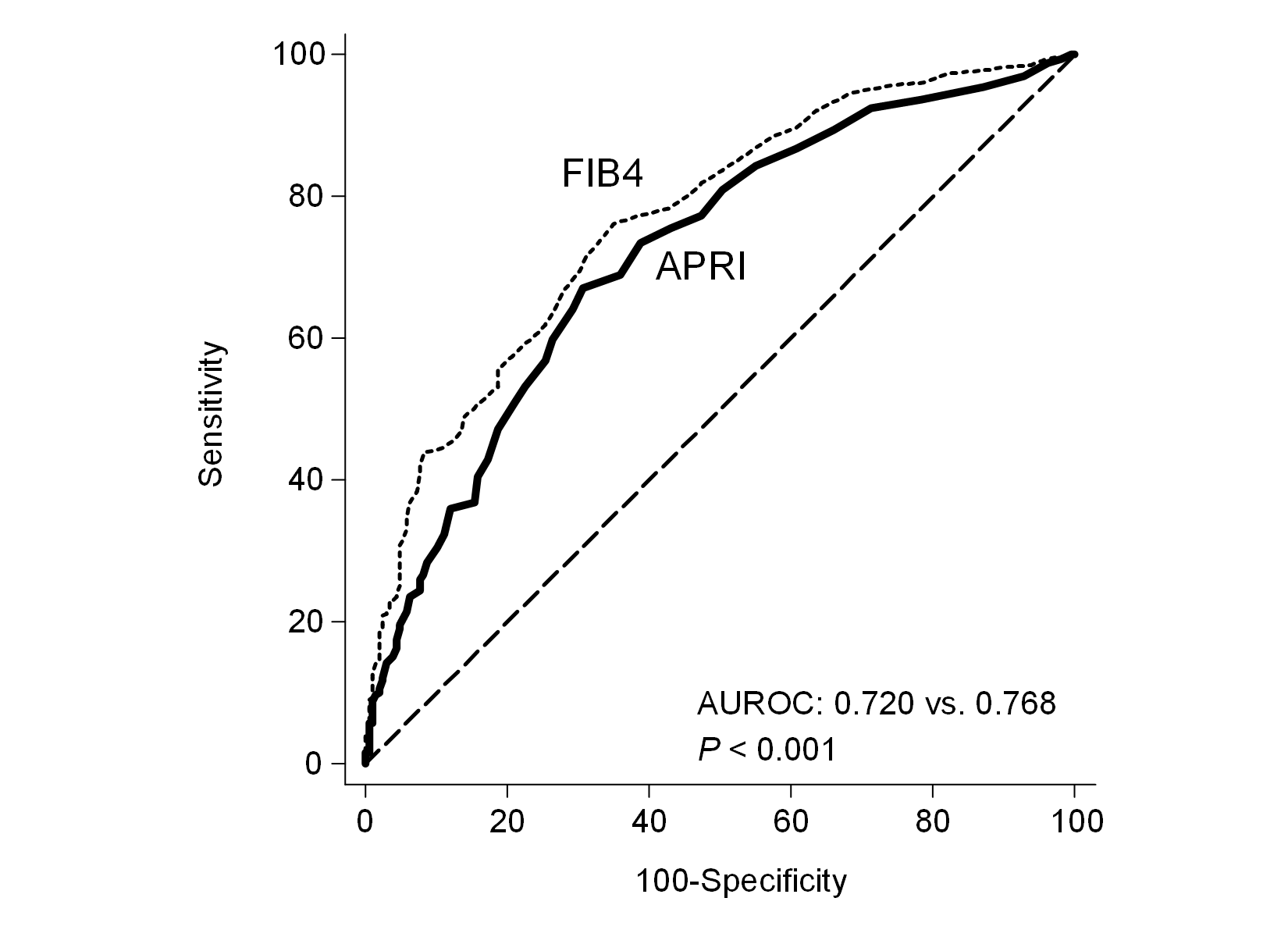

Supplement: S16 Fig — (TIF) [file pone.0199760.s016.tif]

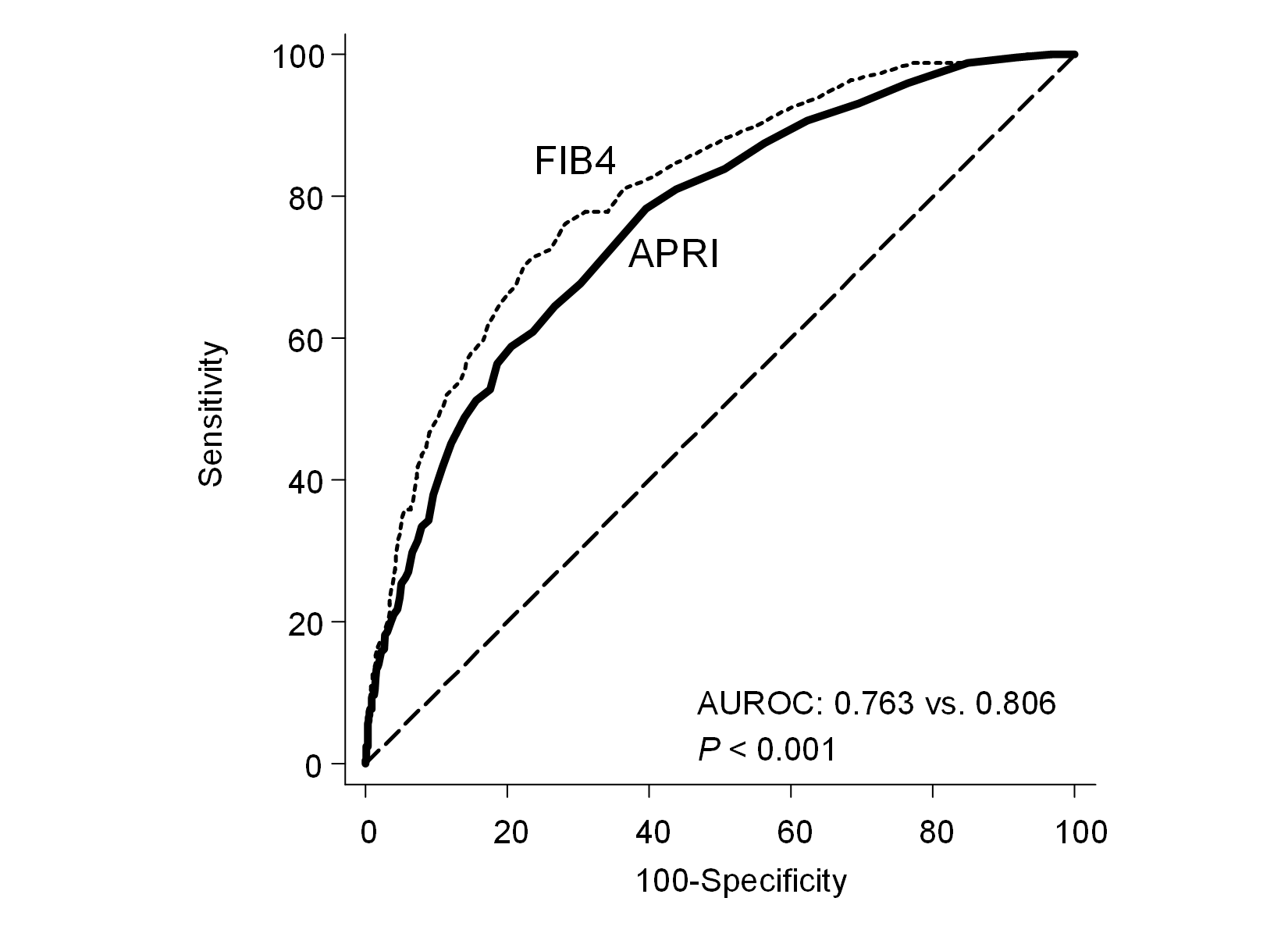

Supplement: S17 Fig — (TIF) [file pone.0199760.s017.tif]

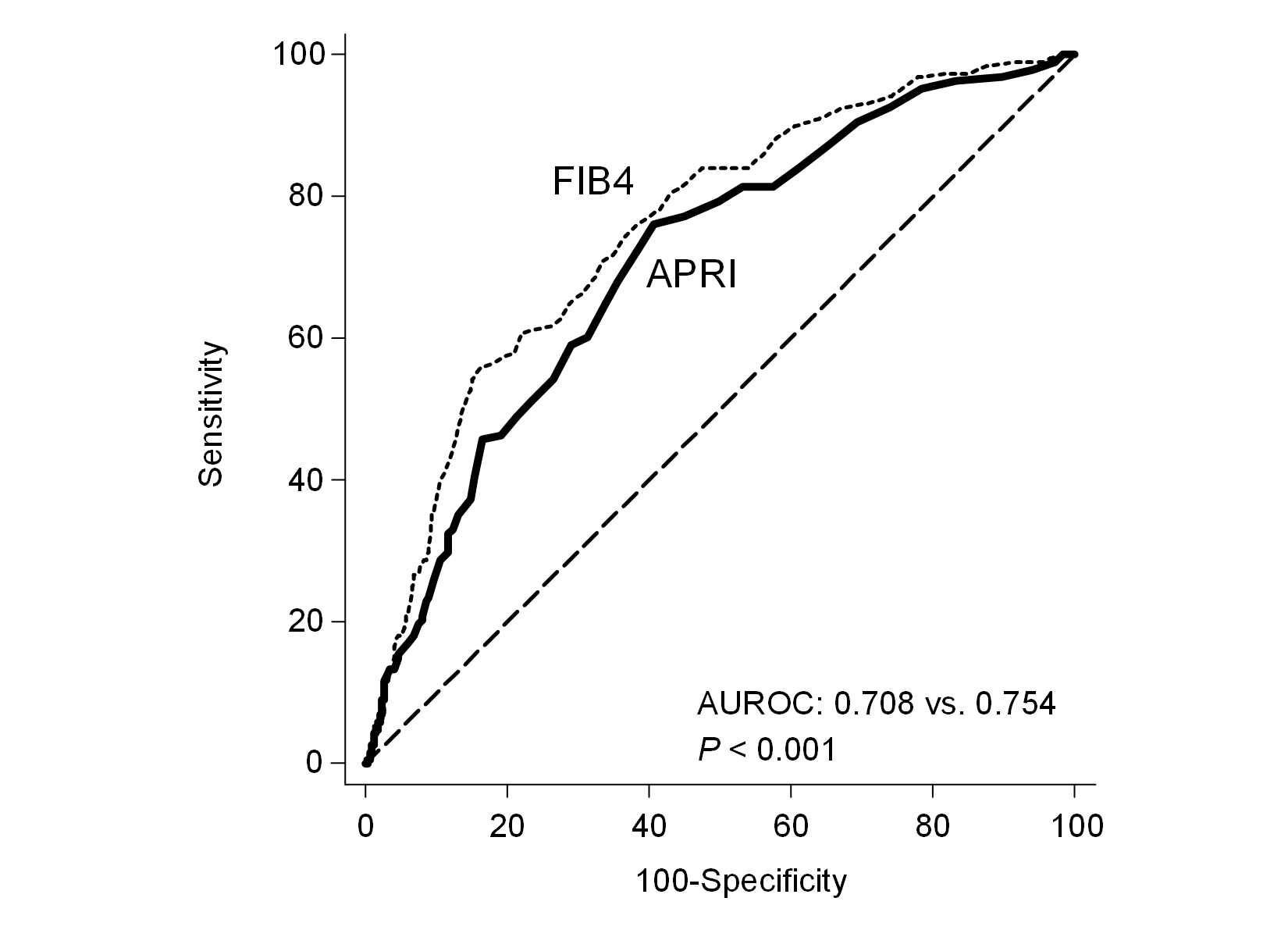

Supplement: S18 Fig — (TIF) [file pone.0199760.s018.tif]
