# Supplementary material for: APRI and FIB-4 in the evaluation of liver fibrosis in chronic hepatitis C patients stratified by AST level
Source: PLoS One. 2018 Jun 28;13(6):e0199760. doi: 10.1371/journal.pone.0199760 (PMC6023204; doi:10.1371/journal.pone.0199760)
Supplement: S1 Table — (DOCX) [file pone.0199760.s019.docx]

Table 1. Comparison of Demographic, Laboratory and Histological Characteristics in Male versus Female Patients.

|  | Male cases (N=913) | female cases (N=803) | *P* value |
| --- | --- | --- | --- |
| Age (yrs) | 54.6 ± 9.5 | 50.9 ± 12.4 | <0.001 |
| BMI (Kg/m^2^) | 24.5 ± 3.9 | 24.7 ± 3.1 | 0.39 |
| Platelet (10^9^/L) <150 | 351 (43.7%) | 329 (36%) | 0.001 |
| AST (IU/L) | 95 (65 - 138) | 87 (58 - 131) | 0.13 |
| ALT (IU/L) | 126 (88 - 187) | 143 (96 - 217) | <0.001 |
| F0 | 137 (17.1%) | 188 (20.6%) | 0.03 |
| F1 | 213 (26.5%) | 270 (29.6%) |  |
| F2 | 71 (8.8%) | 92 (10.1%) |  |
| F3 | 157 (19.6%) | 152 (16.6%) |  |
| F4 | 225 (28%) | 211 (23.1%) |  |

Data were expressed as mean ± SD or median (interquantile). BMI, body mass index; AST, Aspartate Aminotransferase; ALT, Alanine Aminotransferase; F, fibrosis
