# Supplementary material for: APRI and FIB-4 in the evaluation of liver fibrosis in chronic hepatitis C patients stratified by AST level
Source: PLoS One. 2018 Jun 28;13(6):e0199760. doi: 10.1371/journal.pone.0199760 (PMC6023204; doi:10.1371/journal.pone.0199760)
Supplement: S2 Table — (DOCX) [file pone.0199760.s020.docx]

Table 2. Comparison of Demographic, Laboratory and Histological Characteristics in Patients with Age ≥65 years versus Age<65 years.

|  | Non-elderly cases (age<65years, N=1496) | Elderly cases (age ≥65 years, N=220) | *P* |
| --- | --- | --- | --- |
| Age (yrs) | 50.1 ± 9.9 | 69.3 ± 3.7 | <0.001 |
| Male sex (%) | 796 (53.2%) | 117 (53.2%) | 1.0 |
| BMI (Kg/m^2^) | 24.7 ± 3.5 | 24 ± 3.5 | 0.006 |
| Platelet (10^9^/L) <150 | 550 (36.8%) | 130 (59.1%) | <0.001 |
| AST (IU/L) | 89 (59.5 - 133) | 103.5 (75.5 - 152) | 0.002 |
| ALT (IU/L) | 135 (93 - 209) | 125 (93 - 186) | 0.23 |
| F0 | 307 (20.5%) | 18 (8.2%) | <0.001 |
| F1 | 435 (29.1%) | 48 (21.8%) |  |
| F2 | 145 (9.7%) | 18 (8.2%) |  |
| F3 | 255 (17%) | 54 (24.5%) |  |
| F4 | 354 (23.7%) | 82 (37.3%) |  |

Data were expressed as mean ± SD or median (interquantile). BMI, body mass index; AST, Aspartate Aminotransferase; ALT, Alanine Aminotransferase; F, fibrosis
