# Supplementary material for: APRI and FIB-4 in the evaluation of liver fibrosis in chronic hepatitis C patients stratified by AST level
Source: PLoS One. 2018 Jun 28;13(6):e0199760. doi: 10.1371/journal.pone.0199760 (PMC6023204; doi:10.1371/journal.pone.0199760)
Supplement: S3 Table — (DOCX) [file pone.0199760.s021.docx]

Table 3. Comparison of Demographic, Laboratory and Histological Characteristics In Patients With Normal weight, Overweight and Obese

|  | BMI<24, N=687 | BMI 24-27, N=493 | BMI>27, N=355 | *P* |
| --- | --- | --- | --- | --- |
| Age (yrs) | 52.2 ± 12 | 52.9 ± 10.1 | 52.3 ± 10.9 | 0.49 |
| Male sex (%) | 340 (49.5%) | 293 (59.4%) | 185 (52.1%) | 0.003 |
| Platelet (10^9^/L) <150 | 264 (38.4%) | 200 (40.6%) | 144 (40.6%) | 0.70 |
| AST (IU/L) | 91 (61 - 133) | 92 (60 - 135) | 92 (64 - 133) | 0.99 |
| ALT (IU/L) | 130 (91 - 206) | 142 (94 - 218) | 135 (96 - 196) | 0.56 |
| F0 | 138 (20.1%) | 73 (14.8%) | 54 (15.2%) | < 0.001 |
| F1 | 231 (33.6%) | 136 (27.6%) | 88 (24.8%) |  |
| F2 | 62 (9%) | 43 (8.7%) | 42 (11.8%) |  |
| F3 | 120 (17.5%) | 85 (17.2%) | 72 (20.3%) |  |
| F4 | 136 (19.8%) | 156 (31.6%) | 99 (27.9%) |  |

Data were expressed as mean ± SD or median (interquantile). BMI, body mass index; AST, Aspartate Aminotransferase; ALT, Alanine Aminotransferase; F, fibrosis. Patients were categorized as normal weight or underweight (<24 kg/m^2^), overweight (24–27 kg/m^2^), or obese (>27 kg/m^2^) according to the definition of the Health Promotion Administration of the Ministry of Health and Welfare in Taiwan [18].
