# Supplementary material for: APRI and FIB-4 in the evaluation of liver fibrosis in chronic hepatitis C patients stratified by AST level
Source: PLoS One. 2018 Jun 28;13(6):e0199760. doi: 10.1371/journal.pone.0199760 (PMC6023204; doi:10.1371/journal.pone.0199760)
Supplement: S4 Table — (DOCX) [file pone.0199760.s022.docx]

Table 4. Comparison of Diagnostic Accuracies Of APRI For Predicting Liver Fibrosis in Male versus Female patients

| Index | AUROC*_cutoff_* | cutoff | sensitivity*_cutoff_* | specificity*_cutoff_* | PPV*_cutoff_* | NPV*_cutoff_* | Sensitivity + Specificity-1 |
| --- | --- | --- | --- | --- | --- | --- | --- |
| To predict fibrosis ≥2 |  |  |  |  |  |  |  |
| Male | 0.67 (0.64-0.70) | 1.6 | 59.8% | 73.8% | 69.4% | 64.9% | 34.1% |
| Female | 0.69 (0.65-0.72) | 1.4 | 75.3% | 61.7% | 71.8% | 65.9% | 37.0% |
| To predict fibrosis ≥3 |  |  |  |  |  |  |  |
| Male | 0.68 (0.65-0.71) | 1.6 | 64.5% | 71.3% | 59.7% | 75.2% | 36.1% |
| Female | 0.69 (0.66-0.72) | 1.4 | 78.8% | 58.7% | 63.4% | 75.3% | 37.5% |
| To predict fibrosis=4 |  |  |  |  |  |  |  |
| Male | 0.70 (0.67-0.74) | 2.2 | 62.1% | 78.1% | 46.0% | 87.3% | 40.2% |
| Female | 0.70 (0.66-0.73) | 2.2 | 67.6% | 72.2% | 48.6% | 85.1% | 39.7% |

APRI, aspartate aminotransferase (AST)- to-platelet ratio index; AUROC, area under receiver operating characteristic
