# Supplementary material for: APRI and FIB-4 in the evaluation of liver fibrosis in chronic hepatitis C patients stratified by AST level
Source: PLoS One. 2018 Jun 28;13(6):e0199760. doi: 10.1371/journal.pone.0199760 (PMC6023204; doi:10.1371/journal.pone.0199760)
Supplement: S5 Table — (DOCX) [file pone.0199760.s023.docx]

Table 5. Comparison of Diagnostic Accuracies Of FIB-4 For Predicting Liver Fibrosis in Male versus Female patients

| Index | AUROC*_cutoff_* | cutoff | sensitivity*_cutoff_* | specificity*_cutoff_* | PPV*_cutoff_* | NPV*_cutoff_* | Sensitivity + Specificity-1 |
| --- | --- | --- | --- | --- | --- | --- | --- |
| To predict fibrosis ≥2 |  |  |  |  |  |  |  |
| Male | 0.71 (0.68-0.74) | 2.4 | 67.5% | 74.7% | 72.6% | 69.8% | 42.1% |
| Female | 0.69 (0.66-0.72) | 3.0 | 65.6% | 72.6% | 75.6% | 62.0% | 38.1% |
| To predict fibrosis ≥3 |  |  |  |  |  |  |  |
| Male | 0.73(0.70-0.76) | 2.9 | 63.9% | 81.5% | 69.5% | 77.4% | 45.4% |
| Female | 0.72 (0.69-0.75) | 3.7 | 61.5% | 82.4% | 76.1% | 70.2% | 43.9% |
| To predict fibrosis=4 |  |  |  |  |  |  |  |
| Male | 0.74 (0.70-0.77) | 2.9 | 73.0% | 74.4% | 46.1% | 90.2% | 47.4% |
| Female | 0.73 (0.70-0.76) | 3.5 | 75.1% | 70.9% | 50.2% | 88.0% | 46.0% |

FIB-4, fibrosis index based on the four factors; AUROC, area under receiver operating characteristic
