# Supplementary material for: APRI and FIB-4 in the evaluation of liver fibrosis in chronic hepatitis C patients stratified by AST level
Source: PLoS One. 2018 Jun 28;13(6):e0199760. doi: 10.1371/journal.pone.0199760 (PMC6023204; doi:10.1371/journal.pone.0199760)
Supplement: S6 Table — (DOCX) [file pone.0199760.s024.docx]

Table 6. Comparison of Diagnostic Accuracies Of APRI For Predicting Liver Fibrosis in Elderly (age ≥65 years) and Non-elderly (age <65 years) patients

| Index | AUROC*_cutoff_* | cutoff | sensitivity*_cutoff_* | specificity*_cutoff_* | PPV*_cutoff_* | NPV*_cutoff_* | Sensitivity + Specificity-1 |
| --- | --- | --- | --- | --- | --- | --- | --- |
| To predict fibrosis ≥2 |  |  |  |  |  |  |  |
| age ≥65 years | 0.60 (0.53-0.67) | 1.9 | 58.4% | 62.1% | 78.3% | 39.1% | 23.6% |
| Age <65 years | 0.69 (0.66-0.71) | 1.4 | 71.6% | 65.5% | 67.8% | 69.4% | 37.1% |
| To predict fibrosis ≥3 |  |  |  |  |  |  |  |
| age ≥65 years | 0.60 (0.54-0.67) | 2.3 | 51.5% | 69.1% | 72.9% | 46.8% | 24.0% |
| Age <65 years | 0.69 (0.66-0.71) | 1.6 | 66.5% | 70.8% | 61.0% | 75.5% | 38.1% |
| To predict fibrosis=4 |  |  |  |  |  |  |  |
| age ≥65 years | 0.6 (0.52-0.66) | 2.3 | 54.9% | 63.0% | 46.9% | 70.2% | 22.3% |
| Age <65 years | 0.72 (0.69-0.74) | 2.2 | 65.5% | 77.4% | 47.4% | 87.9% | 43.0% |

APRI, aspartate aminotransferase (AST)- to-platelet ratio index; AUROC, area under receiver operating characteristic
