# Supplementary material for: APRI and FIB-4 in the evaluation of liver fibrosis in chronic hepatitis C patients stratified by AST level
Source: PLoS One. 2018 Jun 28;13(6):e0199760. doi: 10.1371/journal.pone.0199760 (PMC6023204; doi:10.1371/journal.pone.0199760)
Supplement: S7 Table — (DOCX) [file pone.0199760.s025.docx]

Table 7. Comparison of Diagnostic Accuracies Of FIB-4 For Predicting Liver Fibrosis in Elderly (age ≥65 years) and Non-elderly (age <65 years) patients

| Index | AUROC*_cutoff_* | cutoff | sensitivity*_cutoff_* | specificity*_cutoff_* | PPV*_cutoff_* | NPV*_cutoff_* | Sensitivity + Specificity-1 |
| --- | --- | --- | --- | --- | --- | --- | --- |
| To predict fibrosis ≥2 |  |  |  |  |  |  |  |
| age ≥65 years | 0.66 (0.59-0.72) | 5.2 | 53.9% | 77.3% | 84.7% | 41.8% | 32.5% |
| Age <65 years | 0.70 (0.68-0.72) | 2.9 | 58.2% | 82.1% | 76.8% | 65.9% | 40.3% |
| To predict fibrosis ≥3 |  |  |  |  |  |  |  |
| age ≥65 years | 0.67 (0.61-0.73) | 5.2 | 57.4% | 76.2% | 79.6% | 52.5% | 35.0% |
| Age <65 years | 0.73 (0.70-0.75) | 2.9 | 65.2% | 80.3% | 69.4% | 77.1% | 45.5% |
| To predict fibrosis=4 |  |  |  |  |  |  |  |
| age ≥65 years | 0.68 (0.61-0.74) | 5.4 | 64.6% | 70.3% | 56.4% | 77.0% | 34.9% |
| Age <65 years | 0.75 (0.72-0.77) | 3.0 | 73.2% | 76.0% | 48.6% | 90.1% | 49.2% |

FIB-4, fibrosis index based on the four factors; AUROC, area under receiver operating characteristic
