# Supplementary material for: APRI and FIB-4 in the evaluation of liver fibrosis in chronic hepatitis C patients stratified by AST level
Source: PLoS One. 2018 Jun 28;13(6):e0199760. doi: 10.1371/journal.pone.0199760 (PMC6023204; doi:10.1371/journal.pone.0199760)
Supplement: S8 Table — (DOCX) [file pone.0199760.s026.docx]

Table 8. Comparison of Diagnostic Accuracies Of APRI For Predicting Liver Fibrosis in Noraml weight, Overweight and Obese patients.

| Index | AUROC*_cutoff_* | Bootstrap AUROC*_cutoff_* | cutoff | sensitivity*_cutoff_* | specificity*_cutoff_* | PPV*_cutoff_* | NPV*_cutoff_* | Sensitivity + Specificity-1 |
| --- | --- | --- | --- | --- | --- | --- | --- | --- |
| To predict fibrosis ≥2 |  |  |  |  |  |  |  |  |
| BMI<24 kg/m^2^ | 0.67 (0.63-0.70) | 0.67 (0.63-0.70) | 1.3 | 77.7% | 55.3% | 60.0% | 74.2% | 33.0% |
| BMI 24-27 kg/m^2^ | 0.69 (0.65-0.72) | 0.69 (0.65-0.72) | 1.9 | 53.2% | 83.7% | 81.6% | 56.8% | 37.9% |
| BMI>27 kg/m^2^ | 0.72(0.68-0.77) | 0.72 (0.68-0.77) | 1.4 | 74.2% | 70.4% | 79.0% | 64.5% | 44.6% |
| To predict fibrosis ≥3 |  |  |  |  |  |  |  |  |
| BMI<24 kg/m^2^ | 0.66(0.62-0.70) | 0.66 (0.62-0.70) | 1.8 | 60.9% | 71.2% | 55.7% | 75.4% | 33.4% |
| BMI 24-27 kg/m^2^ | 0.68 (0.63-0.72) | 0.68 (0.63-0.72) | 1.6 | 64.3% | 70.6% | 67.7% | 67.4% | 37.2% |
| BMI>27 kg/m^2^ | 0.71 (0.66-0.75) | 0.71 (0.66-0.75) | 1.9 | 59.1% | 82.6% | 75.9% | 68.5% | 45.9% |
| To predict fibrosis=4 |  |  |  |  |  |  |  |  |
| BMI<24 kg/m^2^ | 0.68(0.63-0.72) | 0.68 (0.63-0.72) | 2.3 | 57.4% | 77.9% | 39.0% | 88.1% | 38.0% |
| BMI 24-27 kg/m^2^ | 0.68 (0.64-0.73) | 0.68 (0.63-0.73) | 1.9 | 62.2% | 73.9% | 52.4% | 80.8% | 38.7% |
| BMI>27 kg/m^2^ | 0.77 (0.72-0.82) | 0.77 (0.71-0.82) | 2.2 | 73.7% | 79.3% | 57.9% | 88.7% | 53.0% |

APRI, aspartate aminotransferase (AST)- to-platelet ratio index; AUROC, area under receiver operating characteristic; BMI, body mass index; Patients were categorized as normal weight or underweight (<24 kg/m^2^), overweight (24–27 kg/m^2^), or obese (>27 kg/m^2^) according to the definition of the Health Promotion Administration of the Ministry of Health and Welfare in Taiwan [18].
