# Supplementary material for: APRI and FIB-4 in the evaluation of liver fibrosis in chronic hepatitis C patients stratified by AST level
Source: PLoS One. 2018 Jun 28;13(6):e0199760. doi: 10.1371/journal.pone.0199760 (PMC6023204; doi:10.1371/journal.pone.0199760)
Supplement: S9 Table — (DOCX) [file pone.0199760.s027.docx]

Table 9. Comparison of Diagnostic Accuracies Of FIB-4 For Predicting Liver Fibrosis in Noraml weight, Overweight and Obese patients.

| Index | AUROC*_cutoff_* | Bootstrap AUROC*_cutoff_* | cutoff | sensitivity*_cutoff_* | specificity*_cutoff_* | PPV*_cutoff_* | NPV*_cutoff_* | Sensitivity + Specificity-1 |
| --- | --- | --- | --- | --- | --- | --- | --- | --- |
| To predict fibrosis ≥2 |  |  |  |  |  |  |  |  |
| BMI<24 kg/m^2^ | 0.70 (0.67-0.74) | 0.701 (0.666-0.736) | 2.9 | 65.4% | 74.8% | 69.1% | 71.5% | 40.2% |
| BMI 24-27 kg/m^2^ | 0.71(0.67-0.75) | 0.71 (0.67-0.75) | 2.6 | 66.9% | 75.6% | 78.8% | 62.7% | 42.5% |
| BMI>27 kg/m^2^ | 0.73 (0.68-0.78) | 0.73 (0.68-0.77) | 2.3 | 70.9% | 74.7% | 80.8% | 63.1% | 45.8% |
| To predict fibrosis ≥3 |  |  |  |  |  |  |  |  |
| BMI<24 kg/m^2^ | 0.73 (0.69-0.76) | 0.73 (0.69-0.76) | 2.9 | 72.3% | 73.1% | 61.5% | 81.6% | 45.4% |
| BMI 24-27 kg/m^2^ | 0.71(0.67-0.75) | 0.71 (0.67-0.75) | 2.8 | 64.7% | 77.0% | 72.9% | 69.5% | 43.5% |
| BMI>27 kg/m^2^ | 0.77 (0.73-0.81) | 0.77 (0.73-0.82) | 3.1 | 67.8% | 86.4% | 82.3% | 74.3% | 56.1% |
| To predict fibrosis=4 |  |  |  |  |  |  |  |  |
| BMI<24 kg/m^2^ | 0.74 (0.70-0.77) | 0.74 (0.70-0.77) | 3.0 | 78.7% | 68.2% | 37.9% | 92.8% | 46.9% |
| BMI 24-27 kg/m^2^ | 0.71 (0.67-0.75) | 0.71 (0.67-0.75) | 3.5 | 61.5% | 80.4% | 59.3% | 81.9% | 42.0% |
| BMI>27 kg/m^2^ | 0.79(0.75-0.84) | 0.79 (0.74-0.84) | 3.1 | 81.8% | 76.6% | 57.5% | 91.6% | 59.2% |

FIB-4, fibrosis index based on the four factors; AUROC, area under receiver operating characteristic; BMI, body mass index; Patients were categorized as normal weight or underweight (<24 kg/m^2^), overweight (24–27 kg/m^2^), or obese (>27 kg/m^2^) according to the definition of the Health Promotion Administration of the Ministry of Health and Welfare in Taiwan [18].
