# Supplementary material for: APRI and FIB-4 in the evaluation of liver fibrosis in chronic hepatitis C patients stratified by AST level
Source: PLoS One. 2018 Jun 28;13(6):e0199760. doi: 10.1371/journal.pone.0199760 (PMC6023204; doi:10.1371/journal.pone.0199760)
Supplement: S10 Table — (DOCX) [file pone.0199760.s028.docx]

Table 10. Correlation Between APRI, FIB-4 and Clinical Variables (N=1716).

|  | APRI | |  | FIB-4 | |
| --- | --- | --- | --- | --- | --- |
|  | r | *P* |  | r | *P* |
| Age (yrs) | 0.24 | <0.001 |  | 0.47 | <0.001 |
| AST (IU/L) | 0.76 | <0.001 |  | 0.51 | <0.001 |
| ALT (IU/L) | 0.49 | <0.001 |  | 0.14 | <0.001 |
| Albumin (mg/dL) | -0.34 | <0.001 |  | -0.39 | <0.001 |
| INR | 0.16 | <0.001 |  | 0.16 | <0.001 |
| Bilirubulin (mg/dL) | 0.02 | 0.47 |  | 0.03 | 0.26 |
| Platelet (10^9^/L) <150 | -0.52 | <0.001 |  | -0.63 | <0.001 |
| Necroinflammation score | 0.29 | <0.001 |  | 0.27 | <0.001 |
| Fibrosis score | 0.39 | <0.001 |  | 0.45 | <0.001 |
| BMI (Kg/m^2^) | 0.03 | 0.24 |  | 0.04 | 0.16 |

r: Pearson correlation coefficient

BMI, body mass index; AST, Aspartate Aminotransferase; ALT, Alanine Aminotransferase; INR, international normalized ratio;
