# Supplementary material for: APRI and FIB-4 in the evaluation of liver fibrosis in chronic hepatitis C patients stratified by AST level
Source: PLoS One. 2018 Jun 28;13(6):e0199760. doi: 10.1371/journal.pone.0199760 (PMC6023204; doi:10.1371/journal.pone.0199760)
Supplement: S11 Table — (DOCX) [file pone.0199760.s029.docx]

Table 11. Performance Of AST/ALT Ratio ≥1 In Distinguishing Cirrhotic from Non-cirrhotic Patients

|  | AUROC | sensitivity | specificity | PPV | NPV | Sensitivity + Specificity-1 |
| --- | --- | --- | --- | --- | --- | --- |
| AST<37 IU/L | 0.39 (0.35-0.43) | 0.00% | 77.8% | 0.00% | 94.2% | -22.2% |
| 37<AST≤74 IU/L | 0.56 (0.52-0.60) | 17.1% | 94.5% | 37.8% | 85.3% | 11.6% |
| 74<AST≤148 IU/L | 0.56 (0.54-0.59) | 18.5% | 94.3% | 59.2% | 72.2% | 12.8% |
| AST>148 IU/L | 0.59 (0.55-0.64) | 31.4% | 87.1% | 56.7% | 70.3% | 18.5% |

AST, Aspartate Aminotransferase; PPV, positive predictive value; NPV, negative predictive value
